# Predicting in-hospital mortality in children in low- and middle-income countries: A systematic review and meta-analysis of vital signs and anthropometric measurements

**DOI:** 10.1371/journal.pone.0336233

**Published:** 2025-11-10

**Authors:** Lisanne C. A. Smits, Myrthe Datema, Wieger P. Voskuijl, Moses M. Ngari, Mercy Kumwenda, Job C. J. Calis

**Affiliations:** 1 Amsterdam UMC, location University of Amsterdam, Amsterdam Institute for Global Child Health, Emma Children’s hospital, Amsterdam, The Netherlands; 2 Amsterdam UMC, location University of Amsterdam, Department of Global Health, Amsterdam Institute for Global Health and Development, Amsterdam, The Netherlands; 3 Department of Paediatrics and Child Health, Kamuzu University of Health Sciences, Blantyre, Malawi; 4 Clinical Research Department, KEMRI-Wellcome Trust Research Programme, Kilifi, Kenya; 5 Department of Paediatrics, Kamuzu Central Hospital, Lilongwe, Malawi; 6 Paediatric Intensive Care, Emma Children’s Hospital, Amsterdam University Medical Centres, Amsterdam, The Netherlands; Emory University School of Medicine, UNITED STATES OF AMERICA

## Abstract

**Background:**

In low- and middle-income countries (LMICs), child mortality rates remain substantially higher compared to high-income countries, with many deaths preventable through early recognition of deterioration. This systematic review and meta-analysis investigated predictive values of vital signs and anthropometric measurements for paediatric in-hospital mortality in LMICs.

**Methods:**

A search of publicly available data in PubMed and OVID Embase was conducted in November 2021 and last updated in March 2025. Studies that reported on oxygen saturation; respiratory rate; heart rate; blood pressure; temperature; mid-upper arm circumference (MUAC); and/or weight-for-height z-score (WHZ), and paediatric in-hospital mortality were included. Neonatal and paediatric intensive care unit (PICU) studies were excluded. Data was extracted by two independent authors. Forest plots presented odds ratios (OR) using random effect models. Newcastle Ottawa Scale assessed risk of bias.

**Findings:**

104 out of 21,494 yielded studies were included in descriptive analysis and 75 in meta-analysis, encompassing 255,546 children. Associations with in-hospital mortality were observed in hypoxaemia (OR 5.53, 95% CI 4.18–7.30), tachypnoea (OR 1.65, 95% CI 1.16–2.34), tachycardia (OR 1.80, 95% CI 1.22–2.66), bradycardia (OR 3.29, 95% CI 1.38–7.83), hypotension (OR 4.42, 95% CI 2.54–7.70), hyperthermia (OR 1.31, 95% CI 1.04–1.66), hypothermia (OR 3.92, 95% CI 2.76–5.58), low MUAC (OR 3.22, 95% CI 2.12–4.91), and low WHZ (OR 3.19, 95% CI 2.47–4.11).

**Interpretation:**

Several vital signs and anthropometric measurements are strongly associated with in-hospital mortality in children. Hypoxaemia demonstrated the highest odds of mortality, followed by hypotension, hypothermia, bradycardia and severe malnutrition. These findings highlight the need for early recognition and targeted interventions for children presenting with these high-risk signs, to improve outcomes in resource-limited settings and stress the need to monitor vital signs.

**Funding:**

None.

## Introduction

Since 2000, child mortality rate has decreased by approximately 52%. Nevertheless, an estimated 4.8 million children under the age of five died in 2023 [1]. With 80%, the vast majority of these deaths occur in sub-Saharan Africa (SSA) and South Asia, where child mortality rates remain substantially higher than in high-income countries (HICs) [2–4]. Most paediatric in-hospital deaths in LMICs happen within 24 hours after admission and have preventable and treatable causes [5–8]. Therefore, early recognition of deterioration may reduce mortality in these countries.

Deranging vital signs are often the first to indicate clinical deterioration [6,9–12]. In HICs, monitoring non-invasive bedside vital signs such as oxygen saturation, respiratory rate (RR), heart rate (HR), blood pressure (BP) and temperature, is commonly used to discriminate between children at high and low mortality risk [13–16]. These are routinely collected both at admission and during hospital stay, and help healthcare workers (HCWs) to stratify the risk and determine the response to treatment. However, the predictive role of these parameters is less clear in LMICs and may be different than in HICs, due to other etiologies, comorbidities, pathophysiology and late presentation [2,17,18]. Malnutrition is especially prevalent in LMICs and contributes to nearly half of deaths under five years old [19]. Therefore, this might be an important predictor of outcome in these settings and may also affect vital signs [20].

Collecting vital signs requires equipment, which is often scarce in the low-resource setting, and valuable time and effort of the often overstrained HCWs. To use these resources as efficiently as possible, it is important to identify which vital signs are most useful for identifying serious illness and impending deterioration.

Therefore, this systematic review investigated the predictive value of vital signs and anthropometric measurements in determining the risk of paediatric in-hospital mortality in LMICs. Moreover, our data can provide a framework for enhancing monitoring strategies or systems for use in LMICs.

## Methods

This systematic review and meta-analysis, without PROSPERO registration, was conducted according to the Preferred Reporting Items for Systematic Reviews and Meta-Analyses guidelines (PRISMA, S1 Table) [21].

### Search strategy

After creating a search strategy in collaboration with a multidisciplinary team including a clinical librarian, a systematic search was conducted in both Medline and OVID Embase. It was first performed on November 18^th^ 2021 and updated on December 25^th^ 2023 and March 24^th^ 2025. Terms regarding low- and middle-income countries, mortality, children and the parameters of our interest were searched. Full search strategies are available in S1 File.

### Study selection

Studies that reported data on at least one of the vital signs hypoxaemia, tachypnoea, bradypnoea, tachycardia, bradycardia, hypertension, hypotension, hyperthermia, hypothermia, or anthropometric measurements mid-upper arm circumference (MUAC) and weight-for-height z-score (WHZ) regarding the outcome all-cause in-hospital mortality were included. The following definitions were used: Hypoxaemia was a decreased blood oxygen content (SpO₂) below the defined cut-off. Tachypnoea and bradypnoea were defined as respiratory rates above and below age-specific cut-offs, respectively. Tachycardia and bradycardia were defined as heart rates above and below age-specific cut-offs, hypertension and hypotension as blood pressures above and below the defined cut-offs, and hyperthermia and hypothermia as body temperatures above and below the respective cut-offs.

Studies were eligible if they included children aged 1 month up to 18 years, presenting or admitted to a hospital in a LMIC, as defined by the World Bank [22]. Studies were excluded if they only included neonates (age < 1 month) or if the setting was limited to the paediatric intensive care unit (PICU). Studies including both neonates and children were not excluded. There was no exclusion based on language, study design or year of publication. Although we extracted the timing of the specific vital sign measurement, no restriction was used concerning the vital sign studied and outcome measured.

After removing duplicates, all yielded studies were uploaded in ‘Rayyan’ for screening on title and abstract, which was done by two independent reviewers (LS, MD). Full texts of the remaining articles were separately screened by the same two reviewers for final inclusion. Discrepancies were resolved through discussion, or if necessary with a third reviewer (JC).

### Data extraction

Data extraction was performed by one author and checked by a second author. For all included articles, the following study characteristics were extracted: author, title, year of publication, study country, PubMed identifier (PMID), study design, study duration, hospital setting (emergency department (ED); paediatric ward), study population characteristics, sample size, number of events, measured parameters and outcome (in-hospital mortality). For each parameter, odds ratios (OR) on in-hospital mortality were extracted, preferably including raw data for calculating the OR as well as cut-off values. If ORs were not reported, other ratio measures were extracted. The primary outcome was in-hospital mortality.

### Data analysis

For each individual vital sign or anthropometric measurement, forest plots were created using Review Manager 5.4 (RevMan). Forest plots per subgroups, based on their admission diagnosis (e.g., pneumonia), as well as overview plots were provided, and meta-analyses were performed. Additional subgroup analyses comparing different cut-off values were performed regarding oxygen saturation. Heterogeneity was quantified using I². Data was analysed using random effect models using the DerSimonian and Laird method. Raw data was used to calculate odds ratios [23]. Studies not presenting raw data for calculating odds ratios were included in descriptive analysis. Results of vital signs and anthropometric measurements of interest were discussed following the Airway, Breathing, Circulation, Disability, Environment (ABCDE) approach. Grading of Recommendations Assessment, Development and Evaluations (GRADE) assessment per parameter was performed by two independent authors (LS, MD) to assess the quality of evidence.

### Risk of bias assessment

The Newcastle Ottawa Scale (NOS) was used to assess the risk of bias of all included studies on three domains: selection, comparability, and (cohort) outcome or (cross sectional) exposure. Each domain contained one to four questions, all having different answer options with the rating of either “star” or “slash”. Total number of stars per domain per study was assessed, giving an overview of the likelihood of bias per domain. The maximum score was ten. Scores were classified as high (7–9), indicating low risk of bias; moderate (4–6), indicating moderate risk of bias; or low (0–3), indicating high risk of bias. Funnel plots were created to assess publication bias.

## Results

### Study selection

**Fig 1:**
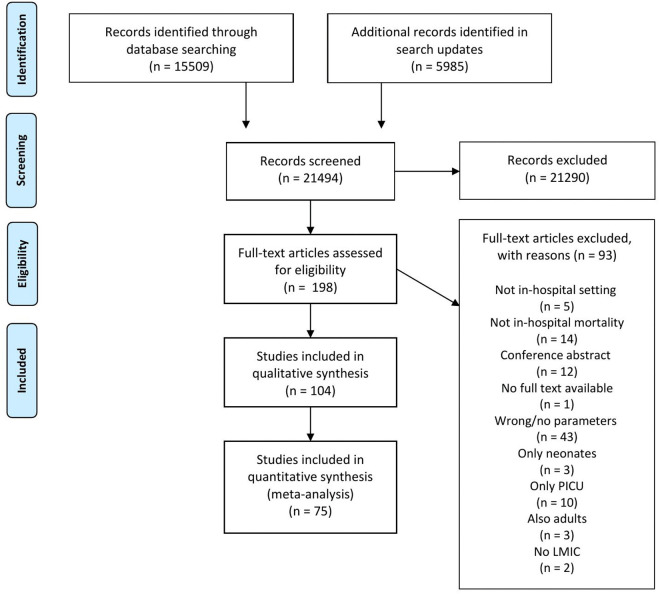
Flowchart of systematic search of the literature according to the PRISMA template. From: Moher D, Liberati A, Tetzlaff J, Altman DG, The PRISMA Group (2009). Preferred Reporting Items for Systematic Reviews and Meta-Analyses: The PRISMA Statement. PLoS Med 6(6): e1000097. https://doi.org/10.1371/journal.pmed1000097.

After removing duplicates, 21,494 articles were screened on title and abstract. Subsequently, 198 full-text articles were assessed for eligibility. A final 104 articles were included in this study (Fig 1), representing 255,546 children. Seventy-five studies presented extractable data and contributed to the meta-analysis.

### Study characteristics

Included studies took place in a range of geographic regions: South Asia (n = 24, 23.1%), Southeast Asia (n = 4, 3.8%), sub-Saharan Africa (n = 67, 64.4%), North Africa (n = 3, 2.9%) and other areas (n = 6, 5.8%). The majority of the studies (n = 94, 90.4%) were conducted in the paediatric ward, while ten studies (9.6%) were conducted in the ED. A detailed description of included studies is presented in Table 1.

**Table 1:** Study characteristics.

Author	Year	Country	Study design	Setting	Population (age)	Outcome parameter	Sample size	Outcome event (n)	Mortality rate (%)
Abdel Baseer [24]	2022	Egypt	Prospective Cohort	Paediatric Ward	Children with nontraumatic coma (1month - 16 years)	In-hospital mortality	137	34	24.8
Abdulkadir [25]	2015	Nigeria	Cross-sectional	Paediatric ward	Children with pneumonia (6–59 months)	In-hospital mortality	200	17	8.5
Abrar [26]	2016	India	Prospective cohort	Paediatric ward	Children presenting with fever of acute onset and who are diagnosed as suspected myocarditis (Mean 6.36 months)	In-hospital mortality	62	11	18
Adegoke [27]	2012	Nigeria	Cross-sectional	Emergency ward	Severely anemic children with venous hematocrit <15% (2–161 months)	In-hospital mortality	311	29	9.3
Adejuyighe [28]	1996	Nigeria	Prospective cohort	Paediatric ward	Children with intra abdominal abscesses (2 months – 15 years)	In-hospital mortality	55	11	20
Agweyu [29]	2018	Kenya	Retrospective cohort	Paediatric ward	Children with pneumonia (2–59 months)	In-hospital mortality	16162	832	5.2
Ahmed Ali [30]	2017	Sudan	Retrospective cohort	Paediatric ward	Children with hemolytic uremic syndrome (4–168 months)	In-hospital mortality	39	5	12.8
Ahmed [31]	2011	Pakistan	Cross-sectional	Paediatric ward	Children with a history of non-traumatic coma (<14 years)	In-hospital mortality	100	29	29
Airlangga [32]	2024	Indonesia	Retrospective Cohort	Paediatric Ward	Children with COVID-19 (1 month – 18 years)	In-hospital mortality	303	9	3.0
Akinbami [33]	2010	Nigeria	Prospective cohort	Emergency ward	All children (12–59 months)	In-hospital mortality	164	11	6.7
Alam [34]	2023	India	Prospective + Retrospective Cohort	Paediatric Ward	Children with MIS-C (Multi-system inflammatory syndrome in children) (<18 years)	In-hospital mortality	98	18	18.4
Alao [35]	2023	Nigeria	Prospective cohort	Paediatric Ward	Children with Acute Kidney Injury (1 month-18 years)	In-hospital mortality	169	25	14.8
Alege [36]	2024	Nigeria	Prospective Cohort	Paediatric Ward	Children with diphtheria (4–7 years)	In-hospital mortality	246	58	23.6
Awasthi [37]	2023	India	Prospective cohort	Paediatric Ward	Children with pneumonia (2–59 months)	In-hospital mortality	6745	93	1.4
Bains [9]	2012	India	Prospective cohort	Emergency department	All children (<18 years)	In-hospital mortality	777	127	16.3
Bashaka [38]	2019	Tanzania	Prospective cohort	Emergency department	Children with undernutrition (1–59 months)	In-hospital mortality	146	4 (<24h); 18 (<30d)	2.9; 12.3
Berkley [39]	2005	Kenya	Prospective cohort	Paediatric ward	All children(12–59 months)	In-hospital mortality	8190	359	4.4
Berkley [40]	2003	Kenya	Prospective cohort	Paediatric ward	All children (>90 days)	In-hospital mortality	8091	436	5
Bokade [41]	2014	India	Prospective cohort	Paediatric ward	Children presented with fever <2 weeks and altered mental status > 4h (1 month – 12 years)	In-hospital mortality	176	34	19.3
Brady [42]	1996	Zimbabwe	Prospective cohort	Paediatric ward	Children with clinical bronchopneumonia (1–6 months)	In-hospital mortality (< 7 days)	40	11	27.5
Briend [43]	1986	Bangladesh	Prospective cohort	Paediatric ward	All admitted (<5 years)	In-hospital mortality	352	34	9.7
Chiabi [44]	2016	Cameroon	Case-control	Paediatric ward	Children with SAM (6–59 months)	In-hospital mortality	106	22	21
Chimhuya [45]	2007	Zimbabwe	Cross-sectional	Paediatric ward	All children (<18 years)	In-hospital mortality	784	250	31.9
Chisti [46]	2012	Bangladesh	Prospective cohort	Paediatric ward	Children with diarrhoea (<5 years)	In-hospital mortality	119	25	21
Chisti [47]	2011−1	Bangladesh	Prospective cohort	Paediatric ward	Children with diarrhoea and pneumonia (<5 years)	In-hospital mortality	198	24	12.1
Chisti [48]	2011−2	Bangladesh	Prospective cohort	Paediatric ward	Children with diarrhoea (0–59 months)	In-hospital mortality	258	29	11.2
Dembele [49]	2019	Philippines	Case-control	Paediatric ward	Children with severe pneumonia (<5 years)	In-hospital mortality	5054	238	4.7
Demers [50]	2000	Central African Republic	Prospective cohort	Paediatric ward	Children with pneumonia (<5 years)	In-hospital mortality	395	49	12.4
Djelantik [51]	2003	Indonesia	Retrospective cohort	Paediatric ward	Children with pneumonia (<2 years)	In-hospital mortality	4351	505	12
Dramaix [52]	1993	Congo	Prospective cohort	Paediatric ward	All children (<18 years)	In-hospital mortality	955	147	15.4
Duke [53]	2001	Papua New Guinea	Prospective cohort	Paediatric ward	Children with clinical symptoms of pneumonia (28 days – 5 years)	In-hospital mortality	151	46	6.5
Eckerle [54]	2022	Malawi	Prospective cohort	Paediatric Ward	Children with severe pneumonia (1–59 months)	In-hospital mortality	884	21	2,4
Ekoube [55]	2024	Cameroon	Retrospective Cohort	Paediatric Ward	Children with bronchiolitis (1–24 months)	In-hospital mortality	215	23	10.7
Fattahi [56]	2022	Iran	Retrospective Cohort	Paediatric Ward	Children with COVID-19 (0–17 years)	In-hospital mortality	645	16	2.5
Fouad [57]	2011	Egypt	Prospective cohort	Emergency department	Children with acute alteration of consciousness (28 days – 12 years)	In-hospital mortality	100	50	50
Gachau [58]	2018	Kenya	Retrospective cohort	Paediatric ward	Children with SAM (1–59 months)	In-hospital mortality	5360	781	15.8
Gallagher [59]	2023	Kenya, Zambia, South Africa, Mali, the Gambia, Bangladesh and Thailand	Retrospective Cohort	Paediatric Ward	Children with pneumonia (2–59 months)	In-hospital mortality	2189	76	3.47
George [60]	2015	Kenya, Uganda, Tanzania	Prospective cohort	Paediatric ward	Severely ill children (2 months – 12 years)	In-hospital mortality	2815	306	10.9
Girum [61]	2018	Ethiopia	Retrospective cohort	Paediatric ward	Children with SAM (<5 years)	In-hospital mortality	400	33	8.25
Girum [62]	2017	Ethiopia	Retrospective cohort	Paediatric ward	Children with SAM (<5 years)	In-hospital mortality	545	51	9.3
Graham [63]	2019	Nigeria	Prospective cohort	Paediatric ward	All children (<15 years)	Secondary: In-hospital mortality	16453	NA	4.2
Gupta [64]	2023	India	Retrospective Cohort	Paediatric Ward	Children (0–18 years)	In-hospital mortality	400	59	14.8
Ikobah [65]	2022	Nigeria	Prospective cohort	Paediatric Ward	Children with SAM (1–60 months)	In-hospital mortality	91	7	7.7
Ilunga-Ilunga [66]	2014	Congo	Prospective cohort	Paediatric ward	Children with severe malaria (<15 years)	In-hospital mortality	1350	80	5.9
Jarso [67]	2015	Ethiopia	Retrospective cohort	Paediatric ward	Severely malnourished children (6–59 months)	In-hospital mortality	947	88	9.3
Jofiro [68]	2018	Ethiopia	Retrospective cohort	Emergency department	All children (7 days – 13 years)	In-hospital mortality	12240	338	27.61
Jung [69]	2009	Korea	Retrospective cohort	Emergency department	Children with severe trauma (<16 years)	In-hospital mortality	102	13	12.7
Kambale [70]	2019	Congo	Retrospective cohort	Paediatric ward	Severely malnourished admitted children (<18 years)	In-hospital mortality	633	58	9.2
Kapoor [71]	2022	India	Case-control	Paediatric Ward	Children with severe pneumonia (2–59 months)	In-hospital mortality	180	17	9.4
Kassaw [72]	2021	Ethiopia	Retrospective cohort	Paediatric ward	Children with SAM (<5 years)	In-hospital mortality	476	54	11.3
Kintwa [73]	2021	Papua New Guinea	Prospective cohort	Paediatric ward	Children with severe malnutrition (7–112 months)	In-hospital mortality	150	19	12.7
Kouéta [74]	2007	Burkina Faso	Case-control	Paediatric ward	Children with severe malaria (0–15 years)	In-hospital mortality	144	72	50
Kumar [75]	2020	India	Prospective cohort	Paediatric ward	All children (1–6 months)	In-hospital mortality	1813	107	5.9
Kumar [76]	2003	India	Prospective cohort	Paediatric ward, (6 PICU)	All children (<18 years)	Ìn-hospital mortality	1099 (99 neonates)	44	4
Kuti [77]	2013	Gambia	Prospective cohort	Paediatric ward	Children with severe pneumonia (2–59 monts)	In-hospital mortality	420	15	3.6
Lazzerini [78]	2016	Malawi	Retrospective cohort	Paediatric ward	Children with pneumonia (0–59 months)	In-hospital mortality	102294	6191	6.1
Lindtjorn [79]	1991	Ethiopia	Retrospective case-control	Paediatric ward	Children with diarrhoea (<5 years)	Ìn-hospital mortality	105	21	20
Macpherson [80]	2019	Kenya	Retrospective cohort	Paediatric ward	Children with pneumonia (5–14 years)	In-hospital mortality	1825	145	7.9
Maitland [81]	2006	Kenya	Retrospective cohort	Paediatric ward	Children with severe malnutrition (>3 months)	In-hospital mortality	920	176	19
Marazzi [82]	2014	Mozambique, Malawi, Guinea	Retrospective cohort	Drug resource enhancement centers	HIV-infected antiretroviral naive children (<15 years)	Ìn-hospital mortality	2215	238	10.7
Mishra [83]	2023	India	Prospective Cohort	Paediatric Ward	Children with shock (1 month – 18 years)	In-hospital mortality	50	10	20.0
Muhanuzi [84]	2019	Tanzania	Prospective cohort	Emergency department	Children with respiratory compromise (<18 years)	In-hospital mortality	165	51	30.9
Mujuru [85]	2012	Zimbabwe	Prospective cohort	Paediatric ward	All children (<18 years)	In-hospital mortality	739	155	21
Muoneke [86]	2011	Nigeria	Cross-sectional	Emergency department	Children with severe anemia (<5 years)	Ìn-hospital mortality	140	19	13.6
Nakubeera- Barungi [87]	2018	Uganda	Prospective cohort	Paediatric ward	Children with SAM (6–59 months)	In-hospital mortality	400	39	9.8
Nantanda [88]	2008	Uganda	Prospective cohort	Paediatric ward	Children with symptoms of severe pneumonia (2–59 months)	In-hospital mortality	157	24	15.3
Nantanda [89]	2014	Uganda	Prospective cohort	Paediatric ward	Children with acute respiratory symptoms (2–59 months)	Ìn-hospital mortality	614	22	3.6
Nasir [90]	2011	Nigeria	Retrospective cohort	Paediatric ward	Admitted children who underwent surgery for TIP (3–15 years)	In-hospital mortality	99	16	10.4
Nathoo [91]	1998	Zimbabwe	Retrospective cohort	Paediatric ward	Children with a history of shigella dysenteriae (1 month – 12 years)	In-hospital mortality	312	95	30.4
Ngaboyeka [92]	2023	Congo	Retrospective cohort	Paediatric Ward	Children with SAM (6–59 months)	In-hospital mortality	9969	NA	8
Nguyen [93]	2022	Vietnam	Retrospective cohort	Paediatric Ward	Children with severe COVID-19 (1 month – 18 years)	In-hospital mortality	96	18	3.2
Njuguna [94]	2019	Kenya	Retrospective cohort	Paediatric ward	HIV-infected, ART-naïve children (0–12 years)	In-hospital mortality	181	39	22
Ochora [95]	2024	Uganda	Prospective Cohort	Paediatric Ward	All acutely ill children (1–59 months)	In-hospital mortality	208	16	7.7
Odeyemi [96]	2021	Nigeria	Prospective cohort	Paediatric ward	Children with clinical and radiological pneumonia (1–60 months)	In-hospital mortality	129	13	10.15
Olson [97]	2013	Malawi	Case-control	Paediatric ward	All children (<15 years)	Ìn-hospital mortality	215	54	33.5
Olupot- Olupot [98]	2020	Uganda	Prospective cohort	Paediatric ward	Children with severe p.falciparum malaria (2 months – 12 years)	In-hospital mortality	662	63	9.5
Orimadegun [99]	2014	Nigeria	Cross-sectional	Paediatric ward	Children with severe p.falciparum malaria (6–59 months)	In-hospital mortality	369	30	8.1
Pannell [100]	2014	Afghanistan	Retrospective cohort	Paediatric ward	Paediatric trauma patients (<18 years)	Ìn-hospital mortality	263	20	8
Rahman [101]	2021	Bangladesh	Retrospective cohort	Paediatric ward	Children with WHO-defined severe pneumonia (2–59 months)	In-hospital mortality	2646	198	7.5
Ramakrishna [102]	2012	Malawi	Prospective cohort	Paediatric ward	Children with WHO defined severe or very severe pneumonia (2 months – 14 years)	In-hospital mortality	233	25	10.7
Roy [103]	2011	Bangladesh	Case-control	Paediatric ward	Severely-malnourished children with diarrhoea (<18 years)	Ìn-hospital mortality	206	103	50
Sachdeva [104]	2016	India	Cross-sectional	Emergency department	All children (6 months – 5 years)	In-hospital mortality	1663	124	7.46
Samuel [105]	2014	Malawi	Retrospective cohort	Paediatric ward	Children who underwent surgery (0–17 years)	In-hospital mortality	1077	80	7
Schellenberg [106]	1999	Tanzania	Retrospective cohort	Paediatric ward	Children with malaria (0–15 years)	Ìn-hospital mortality	2432	72	3
Shafaei [107]	2023	Iran	Case-control	Paediatric Ward	Children with COVID-19 (<18 years)	In-hospital mortality	191	24	12.6
Shah [108]	2016	Sierra Leone	Retrospective cohort	Ebola management centre	Children with laboratory confirmed Ebola virus (<15 years)	In-hospital mortality	91	52	57.1
Shahunja [109]	2013	Bangladesh	Prospective cohort	Special Care Ward of Dhaka Hospital (diarrhoeal treatment center)	Diarrhoeal children with clinical sepsis (0–59 months)	Ìn-hospital mortality	151	23	15.2
Shahunja [110]	2020	Bangladesh	Retrospective cross-sectional	Paediatric ward	Children with diarrhoea and bacteremia (<5 years)	In-hospital mortality	401	45	11
Shann [111]	1989	Papua New Guinea	Prospective cohort	Paediatric ward	Children with cough, chest indrawing and any of the following: severe chest indrawing; HR > 160/min with hepatomegaly; failure to feed; bronchial breathing; grunting; cyanosis; severe chest roentgenogram changes; or a total white blood cell count >30000 cells/ul (>28 days)	In-hospital mortality	748	110	15
Sharma [112]	2021	India	Retrospective cohort	Paediatric ward	Children with confirmed SARS-CoV-2 (<18 years)	In-hospital mortality	100	27	27
Sigauque [113]	2009	Mozambique	Prospective cohort	Paediatric ward	Children with severe pneumonia (0–23 months)	In-hospital mortality	685	76	11.1
Smyth [114]	1997	Zambia	Prospective cohort	Paediatric ward	Children with pneumonia (severe pneumonia or pneumonia with comorbidity, e.g., malnutrition (4 weeks – 5 years)	In-hospital mortality	158	23	14.6
Spooner [115]	1998	Papua New Guinea	Prospective cohort	Paediatric ward	Children with pneumonia (<5 years)	In-hospital mortality	897	62	6.91
Sturgeon [116]	2023	Zimbabwe, Zambia	Prospective Cohort	Paediatric Ward	Children with SAM (0–59 months)	In-hospital mortality	745	70	9.4
Sylla [117]	2015	Senegal	Retrospective cohort	Paediatric ward	All children (0–59 months)	In-hospital mortality	393	39	10
Talabi [118]	2014	Nigeria	Retrospective cohort	Paediatric ward	Children managed for typhoid ileal perforations (<15 years)	In-hospital mortality	45	9	20
Talbert [119]	2009	Kenya	Prospective cohort	Paediatric Ward	Children with severe malnutrition (>6 months)	In-hospital mortality	667	125	19
Talbert [120]	2012	Kenya	Prospective descriptive	Paediatric Ward	Children with SAM(6 months – 12 years)	In-hospital mortality	592	122	20.6
Talbert [121]	2019	Kenya	Retrospective cohort	Paediatric Ward	Children with diarrhoea (2–59 months)	In-hospital mortality	2626	121	4.6
Tette [122]	2016	Ghana	Case-control	Paediatric Ward	All children (<5 years)	In-hospital mortality	240	120	50
Tuti [123]	2017	Kenya	Retrospective cohort	Paediatric Ward	Children with a clinical diagnosis of pneumonia (2–59 months)	In-hospital mortality	10687	252	2.36
Van den Broek [124]	2005	Bangladesh	Case-control	Paediatric Ward	Children with shigellosis (<4 years)	In-hospital mortality	200	100	50
Waller [125]	1995	Gambia	Prospective cohort	Paediatric Ward	Children with severe falciparum malaria (mean 4.4 years)	In-hospital mortality	180	27	15
Wen [126]	2021	Kenya, Malawi	Prospective cohort	Paediatric Ward	Children with SAM (6–156 months)	In-hospital mortality	780	127	16.3

*NA, not available.*

### Data analysis

**Fig 2:**
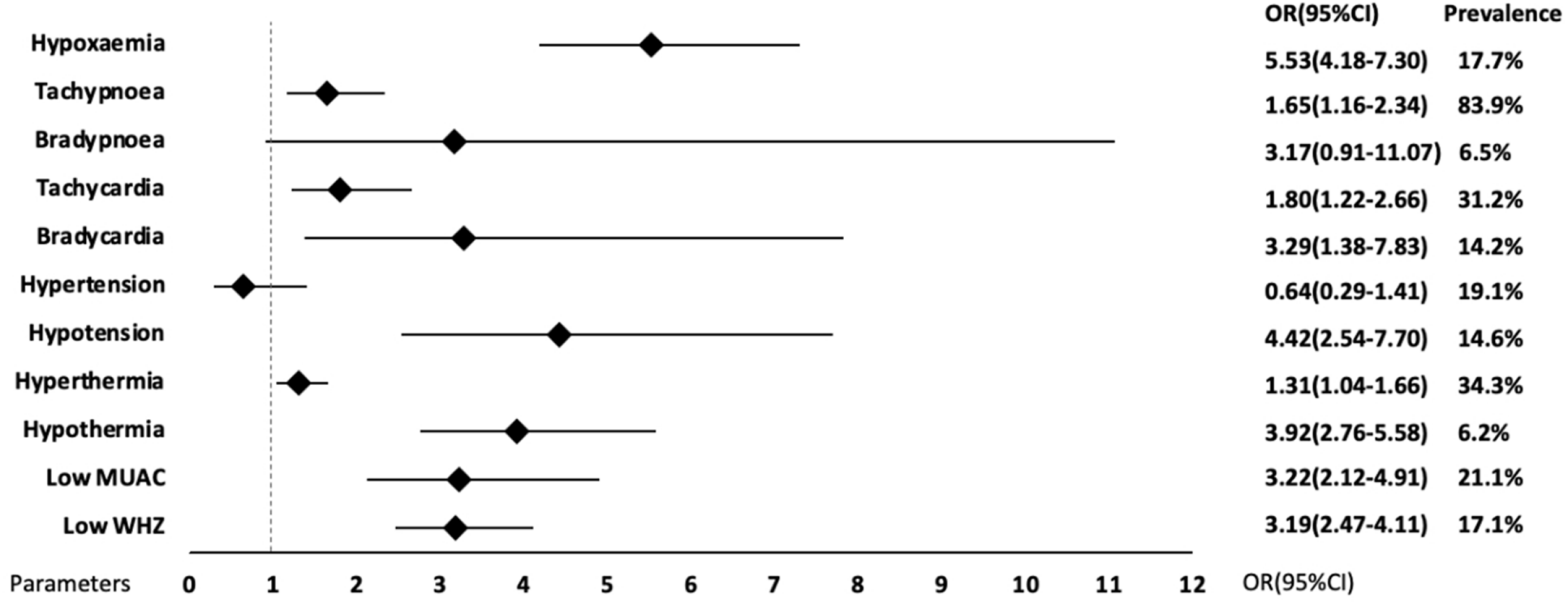
Overview forest plot of abnormal vital signs and anthropometric measurements compared to control on in-hospital mortality. OR, Odds Ratio; CI, Confidence Interval.

Hypoxaemia, tachypnoea, tachycardia, bradycardia, hypotension, hyperthermia, hypothermia, MUAC and WHZ showed associations with paediatric in-hospital mortality, whereas bradypnoea and hypertension showed no association. A summary of the statistics is provided in Fig 2. Included studies used various cut-off values for each parameter. All extracted data are presented in S2 Table, including cut-off values and raw data for odds calculation.

#### Breathing.

**Fig 3:**
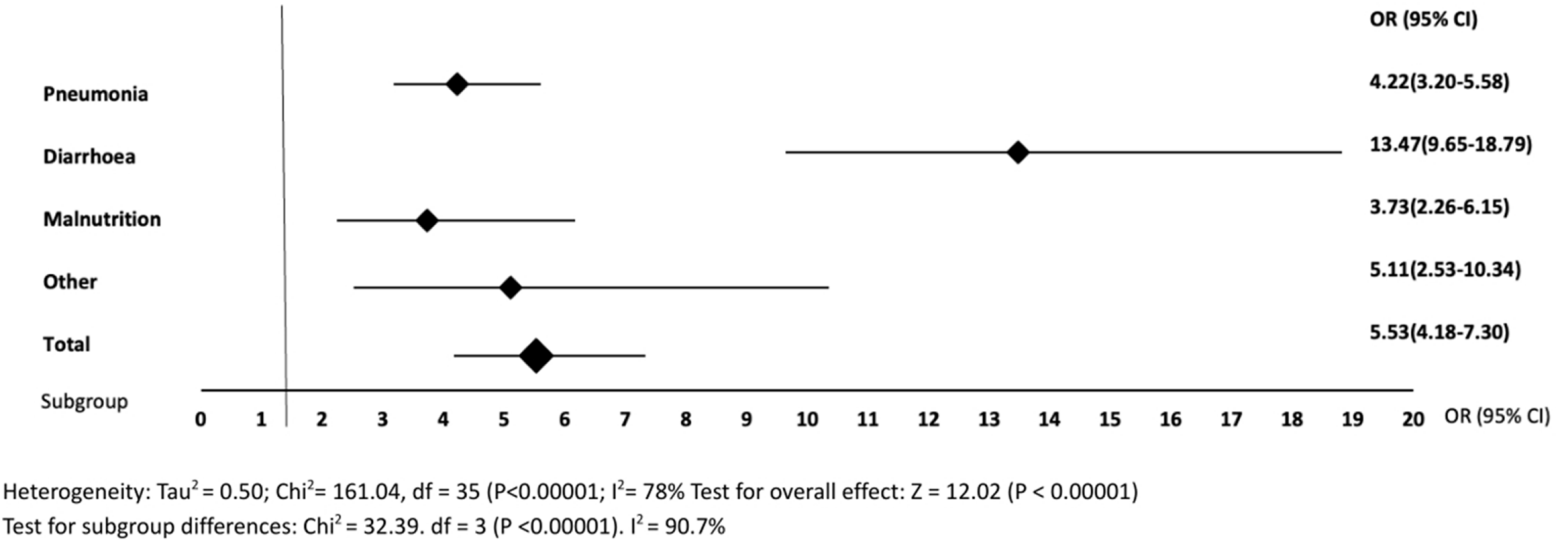
Forest plots of hypoxaemia compared to control on in-hospital mortality. OR, Odds Ratio; CI, Confidence Interval.

Respiratory parameters on in-hospital mortality were hypoxaemia (Fig 3), and tachy- and bradypnoea (S1 Fig). Of studies examining hypoxaemia, 86.0% (37 out of 43) found an association. The pooled results from 36 (83.7%) studies demonstrated an almost six times higher odds of in-hospital mortality (OR 5.53, 95% CI 4.18–7.30, I² 78%) if hypoxaemia was present (Fig 3). The prevalence of hypoxaemia was 17.7%. Cut-offs ranged from 70% to 95% (S2 Table). Subgroup analysis consistently showed associations between hypoxaemia and mortality, particularly pronounced in diarrhoea and other populations. Lower cut-off values were associated with higher odds of in-hospital mortality in all compared subgroups (pneumonia, malnutrition and other diseases) (S1b Fig).

Thirty-four studies reported on tachypnoea and outcome of which 18 (52.9%) reported an association with mortality (S1c Fig). Meta-analysis of 24 studies showed an OR of 1.65 for mortality in children with tachypnoea (95% CI 1.16–2.34, I² 93%). The prevalence of tachypnoea was 83.9%.

Four out of eight studies (50%) that reported on bradypnoea found an association with mortality (S1d Fig). The pooled results of five studies showed no association (OR 3.17, 95% CI 0.91–11.07, I² 74%, prevalence 6.5%). For tachypnoea and bradypnoea, used cut-offs were age-specific and ranged from >16–80/min and <8–40/min, respectively (S2 Table).

#### Circulation.

Circulatory parameters are displayed in S2 Fig. Twenty-one studies reported on tachycardia and outcome. An association was found in 42.9% (n = 9) and the pooled results of 14 studies reported an OR of 1.80 in tachycardic children (95% CI 1.22–2.66, I² 72%) (S2a Fig). Prevalence of tachycardia was 30.0%.

An association between bradycardia and in-hospital mortality was found in eight out of twelve (66.7%) studies. Pooled results of six studies showed an increased odds ratio of 3.29 in bradycardic children (95% CI 1.38–7.83, I² 84%) (S2b Fig). The prevalence of bradycardia was 14.2%. Age-specific cut-offs were used to define tachycardia, ranging from >101 to >220/min, and bradycardia, ranging from <50 to <100/min (S2 Table).

In the meta-analysis of four studies, no association was found between hypertension and in-hospital mortality (OR 0.64, 95% CI 0.29–1.41, I² 3%, prevalence 19.1%) (S2c Fig).

Six out of 13 (46.2%) studies reporting on hypotension found an association with in-hospital mortality. Pooled results of ten studies showed an OR of 4.42 (95% CI 2.54–7.70, I² 52%) (S2d Fig). The prevalence of hypotension was 14.6%. Cut-offs for hypotension using systolic blood pressures were <100 mmHg for infants and <117 mmHg for children above 12 years old (S2 Table).

#### Environment.

Temperature forest plots are presented in S3 Fig. Thirteen out of 49 studies (26.5%) reporting on hyperthermia and outcome, found an association. Besides, 4 out of 49 studies (8.2%) were inversely associated with mortality. Pooled results of 40 studies showed an OR of 1.31 with in-hospital mortality in hyperthermic children (95% CI 1.04–1.66, I² 87%) (S3a Fig). Cut-offs ranged from temperatures >37.5 to >40.0 degrees Celsius (S2 Table). The prevalence of hyperthermia was 34.3%.

An association between hypothermia and in-hospital mortality was found in 70.8% of the studies (17 out of 24). Pooled results of 17 studies showed an OR of 3.92 (95% CI 2.76–5.58 I² 46%) (S3b Fig). The prevalence of hypothermia was 6.2%. Hypothermia cut-offs ranged from <35.0 to <37.0 degrees Celsius (S2 Table).

#### Anthropometric measurements.

Forest plots of anthropometric measurements are presented in S4 Fig. Fourteen out of 22 studies (68.2%) reporting on MUAC, and 17 out of 23 studies (73.9%) reporting on WHZ, found an association with in-hospital mortality. Pooled results showed ORs of 3.22 for MUAC (95% CI 2.12–4.91, I² 88%, 15 studies, prevalence 21.1%) (S4a Fig) and 3.19 for WHZ (95% CI 2.47–4.11, I² 57%, 18 studies, prevalence 17.1%) (S4b Fig). The most common cut-off for low MUAC was < 11.5 cm and cut-offs for WHZ ranged from −2 to −3 SD (S2 Table).

### Risk of bias

Results of the risk of bias assessment are shown in S3 Table. Ninety studies (86.5%) had low risk of bias, while 14 studies (13.5%) had moderate risk of bias. No high risk of bias was observed. Funnel plots are available in S5 Fig and show no major signs of publication bias. According to the GRADE assessment, the found evidence was of low-moderate quality (S4 Table).

## Discussion

This systematic review and meta-analysis is the largest assessment of the predictive value of vital signs and anthropometric measurements on in-hospital mortality in children in LMICs. We found evidence that hypoxaemia is the strongest predictor of in-hospital mortality, followed by hypotension, hypothermia, and bradycardia. Hyperthermia, tachypnoea and tachycardia, were more common, yet less strongly associated. Low values of static anthropometric measurements MUAC and WHZ also proved to be good predictors on admission. No association was found between bradypnoea or hypertension and in-hospital mortality.

In our analysis, hypoxaemia was highly prevalent (17.7%) among all included children and was found to be a major predictor of in-hospital mortality, independent of admission diagnosis with increased odds of almost six. In sensitivity analysis we found that in children with pneumonia, hypoxaemia was associated with mortality (OR 4.22), which is in line with a recent systematic review by Lazzerini et al, showing an increased odds of death (OR 5.47) in hypoxaemic children with acute lower respiratory infections [127]. Despite considerable improvement in child mortality in LMICs, pneumonia is still the leading cause of under-five mortality, with more than 700,000 under-five deaths annually. The vast majority is occurring in resource-limited settings and is preventable [128,129]. A systematic review by Rahman et al. showed high prevalence of hypoxaemia (31–47%) in children with pneumonia [130]. Both the high OR and prevalence emphasize the importance of measuring oxygen levels in children with respiratory infections. Surprisingly, an even stronger association between hypoxaemia and outcome was found in children with non-respiratory conditions such as diarrhoea (OR 13.47). Several factors may explain this finding. Hypoxaemia may identify the sickest children, in whom other organ systems are also compromised, or may select cases with multiple etiologies. Moreover, misdiagnosis at admission and undertreatment may lead to hypoxaemia. Irrespective of these various explanations, consistent findings across studies underscore the powerful predictive value of hypoxaemia for in-hospital mortality in all children. Lower cut-off values were associated with higher odds of mortality, regardless of the subpopulation. The majority of the studies used a cut-off of <90%. This could be used as a boundary for intervention in LMICs. This all together, highlights the importance of oxygen saturation monitoring in all children, regardless of their initial diagnosis and indicates that measurement of oxygen saturation should be prioritised in LMICs over other vital signs with lower odds ratios.

With pooled odds ratios of 3.29 or higher, bradycardia, hypotension and hypothermia, were identified as reliable predictors. This finding may appear surprising, given that healthcare professionals typically emphasize heightened vital signs such as fever and tachycardia during clinical assessment. These decreased vital signs appear to be stronger predictors. However, they were less common than the increased vital signs (6.2–14.6% vs 31.2–83.9%). The importance of decreased vital signs as recognized predictors is well established as they are components of the widely utilized Paediatric Early Warning Score (PEWS) and PEWS for resource-limited settings (PEWS-RL) to identify children at risk for deterioration. However, they are not included in the WHO ETAT flowcharts [8,131,132]. A comprehensive study by Chapman et al. conducted in the United Kingdom compared 18 different PEWS and found that bradycardia was included in 100% of the PEWS and hypotension in 61% of the investigated PEWS [16].

The more prevalent increased vital signs tachypnoea, tachycardia and hyperthermia had only moderate predictive value, with odds ratios ranging from 1.31 to 1.80. According to the World Health Organization (WHO) guidelines, respiratory distress and tachycardia are being reported as emergency signs that require immediate treatment to avert death. Hyperthermia is flagged as a priority sign indicating the need for prompt assessment of a child [133,134]. All increased vital signs are incorporated in the PEWS and PEWS-RL [131,132]. Chapman et al. found that hyperthermia was included in 39%, tachycardia and tachypnoea in 100% and hypertension in 61% of the 18 evaluated PEWS [16]. Together, these findings stress that both abnormalities should alert HCWs of a poor outcome, acknowledging that low values are more strongly associated with outcome, as they may indicate later stages of deterioration.

In our analysis, abnormally low anthropometric measurements (MUAC and WHZ) were highly prevalent and good predictors of in-hospital mortality (OR 3.22 and 3.19, respectively). Worldwide, nearly half of under-five mortality is linked to any form of undernutrition [125]. In 2022, an estimated 45 million children were wasted (low weight-for-height), subsequently leading to increased risk of death [135]. The WHO indicates severe visible wasting as a priority sign that indicates the need for prompt assessment of a child [133,134]. Our findings are in line with previous studies that repeatedly find associations with mortality, and stress the importance of assessing and improving nutritional status [44,104,136–139].

This systematic review has several strengths. We employed a comprehensive search strategy, identifying 104 studies comprising a total of 255,546 children. Of these, 75 studies were included in the meta-analysis. This study is the first to provide forest plots for vital signs and anthropometric measurements with subgroup analysis. Although looking at isolated vital signs and not taking into account possible confounding factors, this provides clinicians in LMICs an immediate hands-on insight into the best performing predictive parameter. Risk of bias was low in the vast majority of included studies. Lack of adjustment for confounding factors was the most frequent risk of bias. Main limitations were due to the lack of available information and the heterogeneity of the studies, which makes it difficult to generalize the results. Due to the diverse aspect of studies included in this review some elements were not or inconsistently described that would ideally be used in sensitivity analyses: e.g., more detailed analyses of age groups, timing of death, location and resource levels of hospitals. Few studies investigated the predictive value of hypertension. Heterogeneity was caused by differences in populations, variations in clinical setting (e.g., emergency department versus ward, rural versus urban hospitals), diversity in study designs, differences across countries and variability in the timing of parameter measurement (at admission versus within 24–48 hours). Subgroup analyses based on parameter cut-offs, apart from hypoxaemia, as well as age-specific subgroup analyses, were not conducted. Due to the wide variability in cut-offs and age groups within the available data, the resulting subgroups were too small to allow for meaningful analyses. Performing such analyses could however enhance the clinical applicability of the findings in LMICs, where standardized protocols are often lacking.

These shortcomings limit the interpretation of our findings in terms of generalizability and in the sense that we cannot identify ideal cut offs, nor report on specific populations or settings. However, the data do underline which parameters may be most useful to collect in general populations and further highlights the complex context of vital sign measurement in paediatrics both for clinical work and research.

Using GRADE, we assessed the certainty of the evidence to be low to moderate due to high inconsistency because of substantial heterogeneity, indirectness and imprecise results because of lack of data on some of the included parameters.

In LMICs there is an increasing focus on measuring vital signs of sick children to prioritize care and allow timely delivery of critical care interventions. Advanced equipment, electronic health records and continuous monitoring are becoming increasingly available [140,141]. Vital signs and anthropometric measurements are useful to help distinguish severity in children in LMICs in both ED and ward settings. Especially in LMICs, diagnostic facilities (e.g., laboratory testing or imaging) are limited [142–144]. Decisions are often based on just clinical signs, making monitoring bedside parameters even more important. In the absence of clear guidelines on which vital signs to measure for assessing a child’s mortality risk, this review provides guidance on what to determine in a setting with limited resources and a lack of trained personnel. Although our data supports the collection of vital signs and anthropometric measurements on admission, and preferably also on regular intervals during admission, none of the included studies reported on the impact of vital sign monitoring to reduce outcome. If the aim of monitoring is to allow timely interventions, one could argue if intermittent vital sign monitoring is sufficient enough to allow prompt action. Besides the selection of the most prominent vital signs to monitor, these practical issues need to be addressed to improve cost-effective monitoring strategies for these vulnerable critically ill populations. Future research should use a prospective cohort or randomised trial design in order to investigate to what extent monitoring these vital signs and anthropometric parameters can reduce mortality, thereby addressing an important knowledge gap. Moreover, we suggest using standard operating procedures and multivariate analyses as well as subgroup analyses (e.g., cut-off values, age groups, or based on diagnoses), to address the issue of confounding factors.

According to the WHO, pulse oximetry should be used in children who have fast breathing or chest indrawing [145]. Pulse oximetry is a feasible, cheap and non-invasive procedure that can effectively identify children at high risk of in-hospital mortality, regardless of their diagnosis. Clinicians should be aware of the high predictive value of hypoxaemia and therefore, oxygen saturation should be measured on a regular basis in all admitted children and could be considered more important than temperature measurement. Referral or oxygen therapy to manage hypoxaemic children can reduce in-hospital mortality and should be implemented in standard routines in hospitals in LMICs. Ideally, in addition to oxygen saturation, measurements of respiratory rate, heart rate, blood pressure, and temperature should be obtained upon presentation in all patients and results below normal limits should alarm health care workers more than higher readings. This finding should be incorporated in WHO and other guidelines and prediction tools. MUAC and WHZ are good yet relatively static predictors of mortality in all children. Therefore, children with low MUAC and/or WHZ should be monitored closely. Equipment for measuring MUAC and WHZ should be procured to improve triage of all children in LMICs. Given that measuring MUAC only requires a measuring tape and WHZ requires both tape and a scale, and considering their similar predictive values, MUAC may be preferable in low-resource settings.

In conclusion, this systematic review shows that hypoxaemia is the strongest predictor of paediatric in-hospital mortality in LMICS, with an odds ratio of 5.53 in the analysis and a substantial prevalence of approximately one in six cases. Decreased vital signs (bradycardia, hypotension, and hypothermia) exhibit greater odds of mortality than increased vital signs (tachypnoea, tachycardia, and hyperthermia). Therefore, despite their lower prevalence, decreased vital signs warrant serious clinical attention. Although anthropometric measurements represent relatively static parameters, they are nonetheless relevant in predicting in-hospital mortality. Although none of the included studies assessed the impact of vital sign monitoring, this can help to timely start life saving interventions such as administering oxygen. Policymakers should prioritize enhancing access to equipment for measuring vital signs such as pulse oximetry and anthropometric measures in LMIC healthcare settings. Most of these measurements can be obtained at a low cost and do not require advanced equipment. Enhancing access to such tools can facilitate more accurate assessments of mortality risk and are expected to ultimately contribute to the reduction of child mortality. Routine assessment is important to identify those at elevated risk of mortality in LMICs.

## Supporting information

S1 Table PRISMA checklist. (PDF)

S1 File Search strategy. (PDF)

S2 Table Extracted data on in-hospital mortality per vital sign or anthropometric measurement. (PDF)

S1 Fig Forest plots of abnormal respiratory parameters compared to control on in-hospital mortality. (PDF)

S2 Fig Forest plots of abnormal circulatory parameters compared to control on in-hospital mortality. (PDF)

S3 Fig Forest plots of abnormal temperature compared to control on in-hospital mortality. (PDF)

S4 Fig Forest plot of abnormal anthropometric measurements compared to control on in-hospital mortality. (PDF)

S3 Table Risk of bias assessment (Newcastle Ottawa Scale). (PDF)

S5 Fig Funnel plots publication bias. (PDF)

S4 Table GRADE assessment. (PDF)

## Abbreviations

LMIC: low- and middle-income country
MUAC: mid-upper arm circumference
WHZ: weight-for-height z-score
OR: odds ratio
SSA: sub-Saharan Africa
HIC: high-income country
HR: heart rate
RR: respiratory rate
BP: blood pressure
HCW: healthcare worker
PRISMA: Preferred Reporting Items for Systematic Reviews and Meta-Analyses guidelines
PICU: paediatric intensive care unit
PMID: pubMed identifier
ED: emergency department
NOS: Newcastle Ottawa Scale
ABCDE: Airway, Breathing, Circulation, Disability, Environment
GRADE: Grading of Recommendations Assessment, Development and Evaluations
PEWS: Paediatric Early Warning Score
PEWS-RL: Paediatric Early Warning Score for resource-limited settings
WHO: World Health Organization
