# Supplementary material for: Predicting in-hospital mortality in children in low- and middle-income countries: A systematic review and meta-analysis of vital signs and anthropometric measurements
Source: PLoS One. 2025 Nov 10;20(11):e0336233. doi: 10.1371/journal.pone.0336233 (PMC12599941; doi:10.1371/journal.pone.0336233)
Supplement: S1 File — (PDF) [file pone.0336233.s002.pdf]

S1 File. Search strategy

PubMed search strategy

1. (Low-income countries[tiab] OR Low-Middle-income countries[tiab] OR lower-middle-income countries[tiab] OR low/middle-income countries[tiab] OR LMIC[tiab] OR Developing Countries OR Developing Countries[tiab] OR Developing Country[tiab] OR Less-developed country[tiab] OR Less-developed countries[tiab] OR Third-World Country[tiab] OR Third-World Countries[tiab] OR Third-World Nation[tiab] OR Third-World Nations[tiab] OR Underdeveloped Country[tiab] OR Under developed Countries[tiab] OR Developing Nations[tiab] OR Developing Nation[tiab] OR Less-Developed Nation[tiab] OR Less-Developed Nations[tiab] OR Low-Resource Setting[tiab] OR Low-resource settings[tiab] OR Afghanistan OR Afghanistan[tiab] OR Burkina Faso[tiab] OR Burkina Faso OR Burundi OR Burundi[tiab] OR Central African Republic[tiab] OR Chad OR Chad[tiab] OR Congo OR Democratic Republic of the Congo OR Congo[tiab] OR Democratic Republic of the Congo[tiab] OR Eritrea OR Eritrea[tiab] OR Ethiopia OR Ethiopia[tiab] OR Gambia OR Gambia[tiab] OR Guinea OR Guinea[tiab] OR Equatorial Guinea OR Equatorial Guinea[tiab] OR Guinea-Bissau OR Guinea-Bissau[tiab] OR Haiti OR Haiti[tiab] OR Korea OR Korea[tiab] OR Liberia OR Liberia[tiab] OR Madagascar OR Madagascar[tiab] OR Malawi OR Malawi[tiab] OR Mali OR Mali[tiab] OR Mozambique OR Mozambique[tiab] OR Niger OR Niger[tiab] OR Rwanda OR Rwanda[tiab] OR Sierra Leone OR Sierra Leone[tiab] OR Somalia OR Somalia[tiab] OR South Sudan OR South Sudan[tiab] OR Sudan OR Sudan[tiab] OR Syrian Arab Republic[tiab] OR Syria OR Syria[tiab] OR Tajikistan OR Tajikistan[tiab] OR Togo OR Togo[tiab] OR Uganda OR Uganda[tiab] OR Yemen OR Yemen[tiab] OR Algeria OR Algeria[tiab] OR Angola OR Angola[tiab] OR Bangladesh OR Bangladesh[tiab] OR Benin OR Benin[tiab] OR Bhutan OR Bhutan[tiab] OR Bolivia OR Bolivia[tiab] OR Cabo Verde OR Cabo Verde[tiab] OR Cambodia OR Cambodia[tiab] OR Cameroon OR Cameroon[tiab] OR Comoros OR Comoros[tiab] OR Cote D'Ivoire OR Cote D'Ivoire[tiab] OR Djibouti OR Djibouti[tiab] OR Egypt OR Egypt[tiab] OR El Salvador OR El Salvador[tiab] OR Eswatini OR Eswatini[tiab] OR Ghana OR Ghana[tiab] OR Honduras OR Honduras[tiab] OR India OR India[tiab] OR Kenya OR Kenya[tiab] OR Kiribati OR Kiribati[tiab] OR Kyrgyzstan OR Kyrgyzstan[tiab] OR Kyrgyz Republic[tiab] OR Laos OR Laos[tiab] OR Lesotho OR Lesotho[tiab] OR Mauritania OR Mauritania[tiab] OR Micronesia OR Micronesia[tiab] OR Mongolia OR Mongolia[tiab] OR Morocco OR Morocco[tiab] OR Myanmar OR Myanmar[tiab] OR Nepal OR Nepal[tiab] OR Nicaragua OR Nicaragua[tiab] OR Nigeria OR Nigeria[tiab] OR Pakistan OR Pakistan[tiab] OR Papua New Guinea OR Papua New Guinea[tiab] OR Philippines OR Philippines[tiab] OR Sao Tome and Principe OR Sao Tome and Principe[tiab] OR Senegal OR Senegal[tiab] OR Solomon Islands OR Solomon Islands[tiab] OR Sri Lanka OR Sri Lanka[tiab] OR Tanzania OR Tanzania[tiab] OR Timor-Leste OR Timor-Leste[tiab] OR Tunisia OR Tunisia[tiab] OR Ukraine OR Ukraine[tiab] OR Uzbekistan OR Uzbekistan[tiab] OR Vanuatu OR Vanuatu[tiab] OR Vietnam OR Vietnam[tiab] OR West Bank and Gaza[tiab] OR Zambia OR Zambia[tiab] OR Zimbabwe OR Zimbabwe[tiab] OR Belize OR Belize[tiab] OR Indonesia OR Indonesia[tiab] OR Iran OR Iran[tiab] OR Samoa OR Samoa[tiab])
2. (Child OR Child, Preschool OR Minors OR child*[tiab] OR Preschool Child*[tiab] OR kid[tiab] OR kids[tiab] OR minor[tiab] OR minors[tiab] OR juvenile[tiab] OR infant*[tiab] OR Infant)

3. (Mortality OR Child Mortality OR Infant Mortality OR Death Rate[tiab] OR Death Rates[tiab] OR Mortality Rate[tiab] OR Mortality Rates[tiab] OR Mortality[tiab] OR Child mortality[tiab] OR Infant Mortality[tiab] OR Death OR Death[tiab] OR Infant Death OR Infant Death[tiab] OR Fatal Outcome OR Outcome[tiab])

4. (Saturation[tiab] OR Hypoxia OR Hypoxia[tiab] OR Hypoxaemia[tiab] OR oxygen saturation[tiab] OR Heart Rate OR Heart Rate[tiab] OR Heart rate control[tiab] OR pulse rate[tiab] OR Tachycardia OR Tachycardia[tiab] OR Bradycardia OR Bradycardia[tiab] OR Heart Rate Variability[tiab] OR Respiratory rate OR Respiratory rate[tiab] OR Respiration rate[tiab] OR Tachypnea OR Tachypnea[tiab] OR Bradypnea[tiab] OR blood pressure OR Arterial pressure OR Blood pressure[tiab] OR

Systolic pressure[tiab] OR arterial pressure[tiab] OR Hypotension OR Hypotension[tiab] OR Tidal Volume OR Tidal Volume[tiab] OR Hypertension OR Hypertension[tiab] OR Temperature OR Body Temperature OR Temperature[tiab] OR Body temperature[tiab] OR Fever OR Fever[tiab] OR Hyperthermia[tiab] OR Hypothermia[tiab] OR Hypothermia OR vital parameters[tiab] OR clinical signs[tiab] OR Vital Signs OR Vital Signs[tiab] OR Vital Sign[tiab] OR Height-Weight Ratio[tiab] OR Height Weight Ratio[tiab] OR Height Weight Ratios[tiab] OR Mid-Upper Arm Circumference[tiab] OR MUAC[tiab])

5. 1 AND 2 AND 3 AND 4

Embase search strategy

1. Low income country/ or developing country/ or Afghanistan/ or Algeria/ or angola/ or burkina faso/ or burundi/ or Bangladesh/ or benin/ or Bhutan/ or Bolivia/ or belize/ or chad/ or cameroon/ or cape verde/ or central african republic/ or comoros/ or congo/ or cote d'ivoire/ or Cambodia/ or djibouti/ or Egypt/ or el Salvador/ or eswatini/ or eritrea/ or ethiopia/ or gambia/ or guinea/ or guinea-bissau/ or ghana/ or Haiti/ or Honduras/ or india/ or Indonesia/ or iran/ or korea/ or Kiribati/ or kyrgyzstan/ or kenya/ or lesotho/ or liberia/ or loas/ or madagascar/ or malawi/ or mali/ or mozambique/ or Mauritania/ or Federated States of Micronesia/ or Mongolia/ or morocco/ or Myanmar/ or niger/ or Nepal/ or Nicaragua/ or Nigeria/ or Pakistan/ or papua new guinea/ or phillipines/ or rwanda/ or senegal/ or "sao tome and principe"/ or sierra leone/ or Somalia/ or south sudan/ or sudan/ or Timor-Leste/ or somalia/ or Syrian Arab republic/ or Sri lanka/ or Solomon islands/ or Tajikistan/ or tanzania/ or togo/ or tunesia/ or uganda/ or Ukraine/ or Vanuatu/ or Viet nam/ or Uzbekistan/ or zambia/ or Zimbabwe/ or Yemen/
2. (developing countr* or developing world or "low- and middle-income countr*" or "low middle income countr*" or LMIC or Afghanistan or Algeria or angola or burkina faso or burundi or Bangladesh or benin or Bhutan or Bolivia or belize or chad or cameroon or cape verde or central african republic or comoros or congo or cote d'ivoire or Cambodia or djibouti or Egypt or el Salvador or eswatini or eritrea or ethiopia or gambia or guinea or guinea-bissau or ghana or Haiti or Honduras or India or Indonesia or iran or korea or Kiribati or kyrgyzstan or Kyrgyz republic or kenya or lesotho or liberia or loas or Madagascar or malawi or mali or mozambique or Mauritania or Federated States of Micronesia or Mongolia or morocco or Myanmar or niger or Nepal or Nicaragua or Nigeria or Pakistan or papua new guinea or phillipines or rwanda or senegal or "sao tome and principe" or sierra leone or Somalia or south sudan or sudan or Timor-Leste or Somalia or Syrian Arab republic or Sri lanka or Solomon islands or Tajikistan or tanzania or togo or tunesia or uganda or Ukraine or Vanuatu or Viet nam or Vietnam or Uzbekistan or zambia or Zimbabwe or yemen).ti,ab,kw.
3. 1 or 2
4. child/ or juvenile/ or exp infant/ or preschool child/
5. (child* or infant* or preschool or pre-school or kid or kids or juvenil* or minors).ti,ab,kw.
6. 4 or 5
7. exp mortality/ or death/ or fatality/

8. (Mortality or Child Mortality or Infant Mortality or Death Rate or Death Rates or Mortality Rate or Mortality Rates or Mortality or Child mortality or Infant Mortality or Death or Death or Infant Death or Infant Death or Fatal Outcome or Outcome).ti,ab,kw.
9. 7 or 8
10. Oxygen saturation/ or blood oxygen tension/ or hypoxia/ or hypoxemia/ or temperature/ or body temperature/ or critical temperature/ or high temperature/ or low temperature/ or hyperthermia/ or hypothermia/ or heart rate/ or heart rate variability/ or tachycardia/ or bradycardia/ or pulse rate/ or breathing rate/ or tachypnea/ or bradypnea/ or exp blood pressure/ or pulse pressure/ or systolic blood pressure/ or hypertension/ or systolic hypertension/ or hypotension/ or depressed blood pressure/ or elevated blood pressure/ or vital sign/ or parameters/ or arm circumference/ or weight height ratio/
11. (Saturation or Hypoxia or Hypoxia or Hypoxaemia or oxygen saturation or Heart Rate or pulse rate or Tachycardia or Bradycardia or Respiratory rate or Respiration rate or Tachypnea or Bradypnea or blood pressure or Arterial pressure or Systolic pressure or Hypotension or Tidal Volume or Hypertension or Temperature or Body Temperature or Fever or Hyperthermia or Hypothermia or vital parameter* or clinical sign* or Vital Sign* or Height-Weight Ratio* or Mid-Upper Arm Circumference or MUAC).ti,ab,kw.
12. 10 or 11
13. 3 and 6 and 9 and 12
