# Supplementary material for: Predicting in-hospital mortality in children in low- and middle-income countries: A systematic review and meta-analysis of vital signs and anthropometric measurements
Source: PLoS One. 2025 Nov 10;20(11):e0336233. doi: 10.1371/journal.pone.0336233 (PMC12599941; doi:10.1371/journal.pone.0336233)
Supplement: S2 Table — (PDF) [file pone.0336233.s003.pdf]

S2 Table. Extracted data on in-hospital mortality per vital sign or anthropometric measurement

Parameter	Cut-off	Population (illness/symptom/general)	Author	Year	Country	OR	Lower 95% CI	Higher 95% CI	AOR (95% CI)	Other statistics (95% CI)
**Respiratory**										
Hypoxaemia	<90%	Pneumonia	Abdulkadir	2015	Nigeria	61.84	3.66	1044.98		RR 48.1 (2.9-790.0)
Hypoxaemia	< 93%	COVID-19	Airlangga	2024	Indonesia	13.92	3.48	55.62		
Hypoxaemia	NA	Diphtheria	Alege	2024	Nigeria	29.69	11.90	74.08		
Hypoxaemia	<90%	Pneumonia	Awasthi	2023	India	5.73	3.79	8.66	3.82 (2.29-6.36)	
Hypoxaemia	Lower SaO2	Bronchopneumonia	Brady	1996	Zimbabwe	NA	NA	NA		
Hypoxaemia	<90%	Pneumonia	Dembele	2019	Philippines	4.09	3.01	5.55	5.06 (3.67-6.94)	
Hypoxaemia	<90%	Pneumonia	Demers	2000	NA	2.99	1.32	6.75		
Hypoxaemia	<85%	Pneumonia	Djelantik	2003	Indonesia	2.7	2.10	3.50		RR 4.3 (3.7–5.1)
Hypoxaemia	<70%	Pneumonia	Duke	2001	Papua New Guinea	2.46	1.30	4.65		
Hypoxaemia	90-92%	Pneumonia	Eckerle	2022	Malawi	1.73	0.5	5.99		
Hypoxaemia	<94%	Bronchiolitis	Ekoube	2024	Cameroon	0.89	0.37	2.13	1.72 (0.90-3.28)	
Hypoxaemia	<90-92% (<90% SA, Zambia)	Pneumonia	Gallagher	2023	Kenya, Zambia, South Africa, Mali, the Gambia, Bangladesh and Thailand	3.85	2.42	6.12	2.04 (1.07-3.90)	
Hypoxaemia	<90%	General	Gupta	2023	India	6.87	3.66	12.91		
Hypoxaemia	<90%	Pneumonia	Kapoor	2022	India	16.95	3.74	76.92		
Hypoxaemia	<90%	Pneumonia	Kuti	2013	Gambia	3.05	1.05	8.87		
Hypoxaemia	<90%	Pneumonia	Macpherson	2019	Kenya	2.76	1.61	4.76		

Hypoxaemia	<92%	Pneumonia	Nantanda	2008	Uganda	6.07	2.25	16.36		
Hypoxaemia	<90%	Pneumonia	Odeyemi	2021	Nigeria	25.62	3.21	204.48		
Hypoxaemia	<90%	Pneumonia	Rahman	2021	Bangladesh	8.94	1.95	40.99	11.08 (7.28-16.87)	
Hypoxaemia	<90%	Pneumonia	Ramakrishna	2012	Malawi	6.52	2.49	17.07	2.96 (0.94 - 9.29)	
Hypoxaemia	<90%	pneumonia	Sigauque	2009	Mozambique	2.19	1.13	4.25		
Hypoxaemia	<92%	pneumonia	Smyth	1997	Zambia	NA	NA	NA		Regression coefficient: -0,0550
Hypoxaemia	<91%	Respiratory compromised (respiratory distress, respiratory failure, respiratory arrest)	Muhanuzi	2019	Tanzania	3.96	1.95	8.07		RR 2.6 (1.6-4.4)
Hypoxaemia	<92%	Respiratory symptoms (acute asthma + pneumonia)	Nantanda	2014	Uganda	NA	NA	NA		HR 12.2 (1.6-92.0)
Hypoxaemia	<90%	Diarrhoea + Pneumonia	Chisti	2011-1	Bangladesh	11.26	2.57	49.33		
Hypoxaemia	<90%	Diarrhoea	Chisti	2012	Bangladesh	8.98	3.02	26.64		
Hypoxaemia	<90%	Diarrhoea + sepsis	Shahunja	2013	Bangladesh	6.94	2.23	21.59	14.78 (1.38-157.90)	
Hypoxaemia	<90%	Diarrhoea	Shahunja	2020	Bangladesh	14.73	7.35	29.52	4.20 (1.74-10.12)	
Hypoxaemia	<90%	Diarrhoea	Talbert	2019	Kenya	12.79	7.59	21.54		RR 8.86 (6.09-12.89)
Hypoxaemia	<95%	Malnutrition + diarrhoea	Talbert	2012	Kenya	2.24	1.28	3.92	2.6 (1.1-6.1)	
Hypoxaemia	<90%	Malnutrition	Kassaw	2021	Ethiopia	5.55	2.92	10.55		HR 5.85 (3.08-11.1)
Hypoxaemia	<90%	Malnutrition	Kintwa	2021	Papua New Guinea	10.49	3.62	30.39		

Hypoxaemia	<95%	Malnutrition	Maitland	2006	Kenya	2.59	1.44	4.68		
Hypoxaemia	<94%	Malnutrition	Nakubeera-Barungi	2018	Uganda	3.27	1.22	8.76		HR 3.92 (1.42-10.83)
Hypoxaemia	<92%	Malaria	Olupot-Olupot	2020	Uganda	4.6	2.32	9.13	3.64 (1.39–9.52)	
Hypoxaemia	<90%	Malaria	Orimadegun	2014	Nigeria	4.71	2.16	10.27	7.54 (2.80-20.29)	
Hypoxaemia	<90%	Anemia	Adegoke	2012	Nigeria	4.667	1.76	12.38		
Hypoxaemia	lower SpO2	Trauma	Pannell	2014	Afghanistan	NA	NA	NA		
Hypoxaemia	<94%	COVID	Sharma	2021	India	10.37	3.36	31.97		
Hypoxaemia	<90%	General	Bains	2012	India	60.95	25.24	147.18		
Hypoxaemia	<90%	General	Graham	2019	Nigeria	9.20	7.70	11.00	7.3 (6.0-9.0)	
Hypoxaemia	<90%	General	Kumar	2003	India	9.29	4.96	17.38		
Hypoxaemia	85-95%	General	Olson	2013	Malawi	1.76	0.70	4.42		
Tachypnoea	>70/min	Pneumonia	Agweyu	2018	Kenya	0.38	0.31	0.47		RR 2.4 (2.0-3.0)
Tachypnoea	age-specific	Pneumonia	Awasthi	2023	India	3.37	1.56	7.3		
Tachypnoea	2-11 months: >40/min, 12 months: >50/min	Pneumonia	Dembele	2019	Philippines	4.28	2.58	7.12	1.89 (1.15-3.34)	
Tachypnoea	>40/min	Pneumonia	Demers	2000	Central African Republic	1.80	0.98	3.28		
Tachypnoea	>60/min	Pneumonia	Djelantik	2003	Indonesia	NA	NA	NA		RR 1.1 (0.92–1.3)
Tachypnoea	Fast breathing for age	Pneumonia	Eckerle	2022	Malawi	1.19	0.35	4.1		
Tachypnoea	2-11 months >50/min; 11-59 months >40/min	Pneumonia	Lazzerini	2016	Malawi	0.84	0.76	0.94	0.80 (0.67–0.95)	

Tachypnoea	>30/min	Pneumonia	Macpherson	2019	Kenya	1.92	1.13	3.27		
Tachypnoea	>60/min	Pneumonia	Spooner	1998	Papua New Guinea	1.13	0.65	1.98		
Tachypnoea	>70/min	Pneumonia	Tuti	2017	Kenya	2.49	1.91	3.25		
Tachypnoea	>70/min	Cough + chest indrawing	Shann	1989	Papua New Guinea	0.98	0.41	2.35		
Tachypnoea	NA	COVID	Sharma	2021	India	1.60	0.66	3.90		
Tachypnoea	fast breathing or respiratory failure	Malnutrition	Girum	2018	Ethiopia	NA	NA	NA		HR 2.36 (1.64-3.4)
Tachypnoea	NA	Malnutrition	Girum	2017	Ethiopia	NA	NA	NA		HR 4.561 (2.56-7.98)
Tachypnoea	NA	Malnutrition	Jarso	2015	Ethiopia	1.00	0.64	1.57		HR 1.0 (0.6-1)
Tachypnoea	NA	Malnutrition	Kambale	2019	Congo	0.67	0.34	1.33		
Tachypnoea	NA	Malnutrition	Kassaw	2021	Ethiopia	NA	NA	NA		HR 2.98 (1.66-5.35)
Tachypnoea	>60/min	Malnutrition	Kintwa	2021	Papua New Guinea	6.19	1.49	25.61		
Tachypnoea	>40/min	Malnutrition	Maitland	2006	Kenya	1.90	1.12	3.23		
Tachypnea	>2SD normal rate for age	Shock	Mishra	2023	India	1.33	0.24	7.35		
Tachypnoea	<12m >50/min >12m >40/min	Malnutrition	Nakubeera-Barungi	2018	Uganda	2.62	1.23	5.62		HR 2.16 (1.07-4.36)
Tachypnoea	>50 (<1yr) >40 (>=1yr)	Malnutrition	Talbert	2012	Kenya	0.99	0.63	1.55		
Tachypnoea	NA	Malaria	Schellenberg	1999	Tanzania	4.50	1.60	12.90	8.8 (2.6-30.5)	
Tachypnoea	Higher RR	Malaria	Waller	1995	Gambia	NA	NA	NA		
Tachypnoea	infant >60, child >50	General	Bains	2012	India	9.83	6.44	14.99		

Tachypnoea	<12m: >50/min, >1y: >40/min	General	Berkley	2003	Kenya	2.25	1.74	2.92		LR 1.66 (1.42-1.94)
Tachypnoea	NA	General	Jofiro	2018	Ethiopia	2.21	1.00	4.88	2.78 (1.19-6.49)	
Tachypnoea	Infant >60, child >50	General	Kumar	2003	India	1.71	0.86	3.38		
Tachypnoea	>60/min	General	Mujuru	2012	Zimbabwe	1.45	0.52	4.07		
Tachypnoea	<3m: 61-80/min, 3-12m: 51-70/min, 1-4y:41-60/min, 4-12y: 31-40/min, >12y: 16-24/min	General	Olson	2013	Malawi	4.76	2.34	9.70		
Tachypnoea	NA	Anemia	Adegoke	2012	Nigeria	4.63	1.90	11.24		
Tachypnoea	NA	Non-traumatic coma	Ahmed	2011	Pakistan	2.20	0.93	5.19		
Tachypnoea	<1w: >50/min, 1w-1m: >40/min, 1m-1y: >34/min, 1-5y: >22/min, 5-12y: >18/min, >12y: >14/min	Surgical	Samuel	2014	Malawi	0.54	0.15	1.92		
Tachypnoea	>50 (<1yr) >40 (>=1yr)	Diarrhoea	Talbert	2019	Kenya	2.68	1.86	3.86		
Bradypnoea	NA	Malnutrition	Bashaka	2019	Tanzania	0.91	0.11	7.84		RR 0.9 (0.2-3.9)
Bradypnoea	NA	Malnutrition	Jarso	2015	Ethiopia	1.35	0.67	2.73		HR 1.4 (0.7–2.9)
Bradypnoea	NA	Malnutrition	Kambale	2019	Congo	13.25	3.18	55.19		
Bradypnoea	<40/min	Pneumonia	Djelantik	2003	Indonesia	4.60	2.50	8.50		
Bradypnoea	Age specific (NA)	Respiratory compromised	Muhanuzi	2019	Tanzania	65.02	3.75	1128.65		RR 3.9 (2.9-5.0)

Bradypnoea	<3m: 20-29/min, 3-12m: 20-24/min, 1-4y: 15-19/min, 4-12y: 12-19/min, >12y: 8-11/min	General	Olson	2013	Malawi	4.76	2.34	9.70		
Bradypnoea	Lower RR	Trauma	Pannell	2014	Afghanistan	NA	NA	NA		
Bradypnoea	NA	Non-traumatic coma	Ahmed	2011	Pakistan	1.19	0.36	3.95		
**Circulatory**										
Tachycardia	NA	MIS-C	Alam	2023	India	5.26	1.41	19.58		
Tachycardia	<12 months: >180/min, >12 months: >140/min	Pneumonia	Dembele	2019	Philippines	1.08	0.80	1.46	2.09 (1.42-3.07)	
Tachycardia	>160/min	Pneumonia	Eckerle	2022	Malawi	4.46	0.49	40.31		
Tachycardia	>180/min <12m, >140/min 13-24m	Bronchiolitis	Ekoube	2024	Cameroon	1.81	0.65	5.08	0.59 (0.31-1.12)	
Tachycardia	>2SD normal rate for age	Shock	Mishra	2023	India	2.59	0.13	52.07		
Tachycardia	>160/min	Pneumonia	Spooner	1998	Papua New Guinea	0.95	0.54	1.66		
Tachycardia	NA	Malnutrition	Bashaka	2019	Tanzania	4.79	1.71	13.46		RR 3.7 (1.6-8.3)
Tachycardia	NA	Malnutrition	Girum	2018	Ethiopia	NA	NA	NA		HR 4.8 (3-7.5)
Tachycardia	NA	Malnutrition	Girum	2017	Ethiopia	NA	NA	NA		HR 27.54 (12.4-61.2)
Tachycardia	NA	Malnutrition	Jarso	2015	Ethiopia	0.55	0.22	1.39		HR 0.6 (0.2-1.5)
Tachycardia	NA	Malnutrition	Kassaw	2021	Ethiopia	NA	NA	NA		HR 2.23 (1.19-4.16)

Tachycardia	infant >160, child >150	General	Bains	2012	India	3.67	2.38	5.66		
Tachycardia	infant >160, child >150	General	Kumar	2003	India	1.79	0.96	3.34		
Tachycardia	<3m: 151-180/min, 3-12m: 151-170/min, 1-4y: 121-150/min, 4-12y: 111-130/min, >12y: 101-120/min	General	Olson	2013	Malawi	1.16	0.52	2.59	3.92 (1.80-8.51)	
Tachycardia	NA	Non-traumatic coma	Ahmed	2011	Pakistan	0.73	0.30	1.78		
Tachycardia	NA	Acute alteration of consciousness	Fouad	2011	Egypt	10.76	1.31	88.47		
Tachycardia	>220/min	Severely ill	George	2015	Kenya, Uganda and Tanzania	5.10	1.70	15.50		
Tachycardia	NA	Anemia	Muoneke	2011	Nigeria	4.86	1.07	22.01		
Tachycardia	>180 (<1yr) >140 (>=1yr)	Diarrhoea	Talbert	2019	Kenya	1.27	0.87	1.86		
Tachycardia	<1y: >180/min, 1-5y: >140/min, 5-12y: >130/min, >12y: >110/min	Surgical	Samuel	2014	Malawi	0.39	0.05	3.03		
Tachycardia	Higher pulse rate	Malaria	Waller	1995	Gambia	NA	NA	NA		
Bradycardia	NA	Malnutrition	Girum	2018	Ethiopia	NA	NA	NA		HR 4.8 (3-7.5)
Bradycardia	NA	Malnutrition	Girum	2017	Ethiopia	NA	NA	NA		HR 27.54 (12.4-61.2)
Bradycardia	NA	Malnutrition	Jarso	2015	Ethiopia	2.82	1.65	4.82		HR 2.6 (1.6-4.2)

Bradycardia	NA	Malnutrition	Kassaw	2021	Ethiopia	NA	NA	NA		HR 2.23 (1.19-4.16)
Bradycardia	<80/min	Malnutrition	Maitland	2006	Kenya	16.56	5.37	51.06		
Bradycardia	NA	Non-traumatic coma	Ahmed	2011	Pakistan	2.25	0.97	5.21		
Bradycardia	NA	Fever + altered mental status	Bokade	2014	India	3.44	1.58	7.49	5.280 (1.6225-7.1870)	
Bradycardia	NA	Acute alteration of consciousness	Fouad	2011	Egypt	0.43	0.17	1.08		
Bradycardia	<80/min	Severely ill	George	2015	Kenya, Uganda and Tanzania	7.77	3.10	19.70		
Bradycardia	<3m:90-109/min, 3-12m: 80-99/min, 1-4y: 70-89/min, 4-12y: 60-69/min, >12y: 50-59/min	General	Olson	2013	Malawi	1.16	0.52	2.59	3.92 (1.80-8.51)	
Bradycardia	<1m: <100/min, <1y: <90/min	Surgical	Samuel	2014	Malawi	0.39	0.05	3.03		
Bradycardia	<90/min	Diarrhoea	Van den Broek	2005	Bangladesh	11.36	3.31	38.97		
Hypertension	>95th percentile age based	Hemolytic uremic syndrome	Ahmed Ali	2017	Sudan	0.53	0.08	3.56		
Hypertension	NA	Non-traumatic coma	Ahmed	2011	Pakistan	1.40	0.37	5.38		
Hypertension	NA	Acute alteration of consciousness	Fouad	2011	Egypt	1.00	0.14	7.39		
Hypertension	>95th percentile for age	AKI	Alao	2023	Nigeria	0.29	0.08	1.04		

Hypotension	NA	nontraumatic coma	Abdel Baseer	2022	Egypt	12.34	4.62	32.96	3.66 (2.03-6.53)	
Hypotension	NA	Myocarditis	Abrar	2016	India	6.97	1.36	35.67		
Hypotension	<5th percentile for age, gender and height	Anemia	Adegoke	2012	Nigeria	4.63	1.90	11.24		
Hypotension	NA	Non-traumatic coma	Ahmed	2011	Pakistan	2.19	0.93	5.19		
Hypotension	<5th percentile for age and height	MIS-C	Alam	2023	India	4.15	1.40	12.30		
Hypotension	NA	AKI	Alao	2023	Nigeria	3.71	0.22	61.49		
Hypotension	NA	Undernutrition	Bashaka	2019	Tanzania	0.96	0.16	5.90		RR 1.0 (0.3-2.8)
Hypotension	NA	Acute alteration of consciousness	Fouad	2011	Egypt	2.46	1.10	5.49		
Hypotension	Lower SBP	Severe trauma	Jung	2009	Korea	NA	NA	NA		
Hypotension	infant <65, child <75	General	Kumar	2003	India	16.50	5.70	47.75		
Hypotension	<5th percentile for age	Shock	Mishra	2023	India	1.36	0.06	30.64		
Hypotension	lower SBP	Trauma	Pannell	2014	Afghanistan	NA	NA	NA		
Hypotension	1m-1y: <100mmhg, 1-5y: <94mmhg, 5-12y: <105mmhg, >12y: <117 mmhg	Surgical children	Samuel	2014	Malawi	0.29	0.01	6.28		
**Temperature**										
Hyperthermia	>39°C	Pneumonia	Agweyu	2018	Kenya	1.97	1.63	2.39		RR 1.9 (1.6-2.3)
Hyperthermia	NA	COVID-19	Airlangga	2024	Indonesia	0.78	0.16	3.86		

Hyperthermia	>38,5	Diphteria	Alege	2024	Nigeria	1.19	0.55	2.54		
Hyperthermia	>38,5 °C	Pneumonia	Dembele	2019	Philippines	2.15	1.59	2.90	2.5 (1.83-3.38)	
Hyperthermia	>38,5 °C	Pneumonia	Djelantik	2003	Indonesia	NA	NA	NA		RR 0.87 (0.69-1.1)
Hyperthermia	>38,0	Bronchiolitis	Ekoube	2024	Cameroon	1.80	0.73	4.44		
Hyperthermia	NA	COVID-19	Fattahi	2022	Iran	1.65	0.59	4.60		
Hyperthermia	>38 °C	Pneumonia	Lazzerini	2016	Malawi	0.88	0.83	0.94		
Hyperthermia	>38 °C	Pneumonia	Macpherson	2019	Kenya	0.94	0.64	1.38		
Hyperthermia	>38,5	Shock	Mishra	2023	India	1.33	0.24	7.35		
Hyperthermia	>38.3 °C	Pneumonia	Odeyemi	2021	Nigeria	8.38	2.17	32.40	34.24 (2.50-469.82)	
Hyperthermia	NA	COVID-19	Shafaei	2023	Iran	1.82	0.69	4.82		
Hyperthermia	>39 °C	Pneumonia	Sigauque	2009	Mozambique	33.55	10.58	106.38		
Hyperthermia	>38 °C	Pneumonia	Spooner	1998	Papua New Guinea	NA	NA	NA		
Hyperthermia	>39 °C	Pneumonia	Tuti	2017	Kenya	1.98	1.38	2.84		
Hyperthermia	>37,8 °C	Respiratory compromise	Muhanuzi	2019	Tanzania	0.67	0.35	1.30		RR 0.8 (0.5-1.2)
Hyperthermia	>38 °C	Acute respiratory symptoms	Nantanda	2014	Uganda	NA	NA	NA		HR 2.1 (0.6-7.5)
Hyperthermia	NA	COVID	Sharma	2021	India	0.47	0.17	1.31		
Hyperthermia	>38,5 °C	Diarrhoea	Chisti	2011	Bangladesh	0.38	0.13	1.15		
Hyperthermia	>38 °C	Diarrhoea	Lindtjorn	1991	Ethiopia	3.76	1.34	10.56		
Hyperthermia	>38 °C	Diarrhoea + sepsis	Shahunja	2013	Bangladesh	0.73	0.22	2.40		
Hyperthermia	NA	Diarrhoea	Shahunja	2020	Bangladesh	0.57	0.30	1.08		
Hyperthermia	>37,5 °C	Malnutrition	Bashaka	2019	Tanzania	1.57	0.51	4.85		RR 1.5 (0.6-3.5)
Hyperthermia	>37,5 °C	Malnutrition	Gachau	2018	Kenya	1.17	0.97	1.43		

Hyperthermia	>39 °C	Malnutrition	Girum	2017	Ethiopia	27.04	13.34	54.84		HR 20.48 (10.70-39.46)
Hyperthermia	NA	SAM	Ikobah	2022	Nigeria	4.13	0.47	35.87		
Hyperthermia	NA	Malnutrition	Kambale	2019	Congo	0.97	0.33	2.84		
Hyperthermia	>37,5 °C	Malnutrition	Maitland	2006	Kenya	0.82	0.48	1.40		
Hyperthermia	NA	SAM	Sturgeon	2023	Zimbabwe, Zambia	1.24	0.73	2.1		
Hyperthermia	>37,5 °C	Malnutrition	Talbert	2012	Kenya	0.99	0.66	1.49		
Hyperthermia	>40 °C	Malaria	Ilunga-Ilunga	2014	Congo	2.76	1.58	4.81	2.9 (1.5-5.7)	
Hyperthermia	>41 °C	Malaria	Kouéta	2007	Burkina Faso	1.14	0.56	2.35	0.8 (0.7-1.0)	
Hyperthermia	>37,5 °C	Malaria	Olupot-Olupot	2020	Uganda	0.56	0.33	0.94	0.79 (0.40-1.57)	
Hyperthermia	>38 °C	Malaria	Schellenberg	1999	Tanzania	0.60	0.30	1.10		
Hyperthermia	Higher temperature	Malaria	Waller	1995	Gambia	NA	NA	NA		
Hyperthermia	38°C	General	Bains	2012	India	3.88	1.10	4.06		
Hyperthermia	>39 °C	General	Berkley	2003	Kenya	0.63	0.46	0.86		LR 0.75 (0.57-0.97)
Hyperthermia	NA	General	Jofiro	2018	Ethiopia	2.58	1.10	6.05	3.17 (1.28-7.86)	
Hyperthermia	≥37,5 °C	General	Mujuru	2012	Zimbabwe	1.19	0.68	2.06		
Hyperthermia	>39 °C	General	Olson	2013	Malawi	17.01	5.38	53.83		
Hyperthermia	>37,5 °C	General	Tette	2016	Ghana	1.35	0.77	2.35		
Hyperthermia	Higher temperature	Abdominal abscesses	Adejuyighe	1996	Nigeria	NA	NA	NA		
Hyperthermia	100 Fahrenheit (37.8 °C)	Non-traumatic coma	Ahmed	2011	Pakistan	0.50	0.21	1.20		
Hyperthermia	NA	AKI	Alao	2023	Nigeria	1.96	0.63	6.07		
Hyperthermia	NA	Alteration of consciousness	Fouad	2011	Egypt	0.17	0.07	0.43		

Hyperthermia	>38 °C	Typhoid ileal perforation	Nasir	2011	Nigeria	3.36	1.00	11.29		
Hyperthermia	>38,0	Covid-19	Nguyen	2022	Vietnam	0.49	0.11	2.13		
Hyperthermia	>38 °C	Ebola	Shah	2016	Sierra Leone	2.78	1.08	7.14		
Hyperthermia	>38,5 °C	Typhoid ileal perforation	Talabi	2014	Nigeria	2.21	0.50	9.72		
Hypothermia	NA	nontraumatic coma	Abdel Baseer	2022	Egypt	3.1	1.31	7.32	5.58 (1.76-17.92)	
Hypothermia	<36 °C	Pneumonia	Djelantik	2003	Indonesia	NA	NA	NA		RR 2.2 (1.5-3.1)
Hypothermia	<36,5 °C	Pneumonia	Kuti	2013	Gambia	20.28	5.75	71.55		
Hypothermia	<36 °C	Diarrhoea	Chisti	2011	Bangladesh	2.09	0.55	7.88		
Hypothermia	<36 °C	Diarrhoea	Nathoo	1998	Zimbabwe	5.14	2.48	10.73	2.12 (1.33-3.39)	
Hypothermia	NA	General	Ochora	2024	Uganda	3.51	1.17	10.50		
Hypothermia	<35 °C	Diarrhoea + Sepsis	Shahunja	2013	Bangladesh	2.34	0.43	12.87		
Hypothermia	<36 °C	Diarrhoea	Van den Broek	2005	Bangladesh	7.58	2.52	22.78	5.7 (1.5-22.1)	
Hypothermia	<36,5 °C	Malnutrition	Gachau	2018	Kenya	1.24	0.93	1.64		
Hypothermia	<35 °C	Malnutrition	Girum	2017	Ethiopia	NA	NA	NA		HR 20.48 (10.7-39.46)
Hypothermia	<35 °C	Malnutrition	Jarso	2015	Ethiopia	6.28	2.33	16.96		HR 3.9 (1.8-8.4)
Hypothermia	NA	Malnutrition	Kambale	2019	Congo	6.03	2.18	16.67		
Hypothermia	<35 °C	Malnutrition	Maitland	2006	Kenya	2.87	0.60	13.58		
Hypothermia	<36 °C	Malnutrition	Roy	2011	Bangladesh	4.81	2.34	9.90		
Hypothermia	NA	SAM	Sturgeon	2023	Zimbabwe, Zambia	2.65	1.22	5.78		
Hypothermia	<35 °C	Malnutrition	Talbert	2009	Kenya	2.21	0.65	7.45		
Hypothermia	<36 °C	General	Bains	2012	India	3.88	1.10	4.06		

Hypothermia	<36 °C	General	Berkley	2003	Kenya	2.88	1.77	4.67		LR 2.61 (1.52-4.49)
Hypothermia	<36 °C	General	Kumar	2003	India	2.74	1.50	5.10		
Hypothermia	35,0-35,9 °C	General	Olson	2013	Malawi	1.84	0.90	3.75		
Hypothermia	<35,56 °C	Non-traumatic coma	Ahmed	2011	Pakistan	3.10	1.31	7.32		
Hypothermia	NA	Acute alteration of consciousness	Fouad	2011	Egypt	9.33	1.12	77.70		
Hypothermia	≤37 °C	Severely ill	George	2015	Kenya, Uganda and Tanzania	NA	NA	NA	1.99 (1.49-2.69)	
Hypothermia	NA	Anemia	Muoneke	2011	Nigeria	2.25	0.42	12.09		
Hypothermia	Lower temperature	Trauma	Pannell	2014	Afghanistan	NA	NA	NA		
**Anthropometry**										
MUAC	<11.5cm	Malnutrition	Chiabi	2016	Cameroon	7.45	0.95	58.71		AUROC 0.809 (0.709-0.911)
MUAC	<11,5 cm	Pneumonia	Eckerle	2022	Malawi	0.46	0.19	1.10		
MUAC	<3m WAZ <-2SD, >3m MUAC <125mm	Pneumonia	Gallagher	2023	Kenya, Zambia, South Africa, Mali, the Gambia, Bangladesh and Thailand	7.93	4.91	12.80	3.90 (1.97-7.71)	
MUAC	NA	Malnutrition	Girum	2017	Ethiopia	4.48	2.20	9.15		HR 3.643 (1.82-7.28)
MUAC	<11.5cm	Malnutrition	Jarso	2015	Ethiopia	6.39	3.00	13.61		HR 5.3 (2.5-11.0)
MUAC	<11.5cm	Malnutrition	Kambale	2019	Congo	2.34	1.11	4.95	1.91 (1.05-3.50)	
MUAC	<11.5cm	Malnutrition	Kintwa	2021	Papua New Guinea	2.22	0.69	7.10		

MUAC	NA	Malnutrition	Nakubeera-Barungi	2018	Uganda	NA	NA	NA		HR 0.82 (0.64-1.06)
MUAC	<11,5 cm	Malnutrition	Ngaboyeka	2023	Congo	NA	NA	NA		RR 2.2 (1.9-2.6)
MUAC	lower MUAC	Malnutrition	Sturgeon	2023	Zimbabwe, Zambia	NA	NA	NA		HR 1.32 (1.15-1.54)
MUAC	<10cm	Malnutrition	Talbert	2012	Kenya	2.72	1.77	4.17	3.5 (1.9-6.5)	
MUAC at admission	<10,5cm	Malnutrition	Wen	2021	Kenya/Malawi	2.08	1.39	3.10		HR 1.7 (1.18-2.45)
MUAC	NA	General	Akinbami	2010	Nigeria	6.82	1.73	26.92		
MUAC	<11.5cm	General	Berkley	2005	Kenya	8.70	6.97	10.86		
MUAC	lower MUAC	General	Briend	1986	Bangladesh	1.47	1.23	1.79		
MUAC	<115mm	General	Dramaix	1993	Congo	3.51	2.41	5.11		
MUAC	<115 mm	General	Kumar	2020	India	NA	NA	NA		
MUAC	<11,5cm	General	Sachdeva	2016	India	3.78	2.52	5.67	3.689 (2.430-5.600)	AUROC 0.698 (0.650-0.746)
MUAC	<115mm	General	Sylla	2015	Senegal	0.81	0.41	1.59		RR 0.81 (0.44-1.5)
MUAC	Higher MUAC	Diarrhoea	Talbert	2019	Kenya	NA	NA	NA		
MUAC	<115mm	Pneumonia	Macpherson	2019	Kenya	3.81	1.66	8.77		
MUAC	NA	HIV-positive	Marazzi	2014	Mozambique, Malawi, Guinea	NA	NA	NA		HR 1.32 (1.23-1.41)
WHZ	≤2SD	Pneumonia	Demers	2000	Central African Republic	3.18	1.50	6.74		
WHZ	<3SD	Pneumonia	Kuti	2013	Gambia	5.05	1.77	14.40		
WHZ	<2SD	Pneumonia	Kapoor	2022	India	5.27	1.76	15.75		

WHZ	≤2SD	General	Akinbami	2010	Nigeria	1.48	0.30	7.34		
WHZ	≤3SD	General	Berkley	2005	Kenya	4.75	3.67	6.16		
WHZ	NA	General	Briend	1986	Bangladesh	1.04	1.01	1.06		
WHZ	<70%	General	Chimhuya	2007	Zimbabwe	4.01	2.29	7.01	2.63 (1.24-5.56)	
WHZ	<3SD	General	Kumar	2020	India	3.45	2.21	5.39	10.62 (NA)	
WHZ	<3SD	General	Sachdeva	2016	India	2.03	1.38	2.99	2.025 (1.373-2.987)	AUROC 0.541 (0.480-0.602)
WHZ	<3SD	General	Sylla	2015	Senegal	7.58	3.69	15.56		RR 6.8 (3.33-13.8)
WHZ	≤2SD	Anemia	Adegoke	2012	Nigeria	4.61	2.00	10.61		
WHZ	≤3SD	Malnutrition	Chiabi	2016	Cameroon	1.73	0.46	6.50		AUROC 0.649 (0.524-0.774)
WHZ	≤3SD	Diarrhoea	Chisti	2011	Bangladesh	1.53	0.66	3.57		
WHZ	NA	Diarrhoea	Lindtjorn	1991	Ethiopia	8.85	3.03	25.84		
WHZ	NA	HIV-positive	Marazzi	2014	Mozambique, Malawi, Guinea	NA	NA	NA		HR 1.58 (1.43-1.74)
WHZ	<3SD	SAM	Ngaboyeka	2023	Congo	NA	NA	NA		NA
WHZ	≤2SD	HIV-positive	Njuguna	2019	Kenya	3.13	1.42	6.92		HR 1.65 (0.81, 3.37)
WHZ	≤2SD	Malaria	Orimadegun	2014	Nigeria	3.65	1.65	8.05		
WHZ	NA	General	Ochora	2024	Uganda	1.30	0.46	3.63		
WHZ	NA	Malaria	Schellenberg	1999	Tanzania	2.50	1.20	5.20		
WHZ	≤3SD	Diarrhoea + sepsis	Shahunja	2013	Bangladesh	0.94	0.34	2.57		
WHZ	≤3SD	Pneumonia	Sigauque	2009	Mozambique	2.42	1.36	4.30		
WHZ	lower WHZ	Malnutrition	Sturgeon	2023	Zimbabwe, Zambia	NA	NA	NA		HR 1.25 (1.11-1.41)

MUAC, mid-upper arm circumference; WHZ, weight-for-height score; OR, odds ratio; 95% CI, 95% confidence interval; RR, AOR, adjusted odds ratio; RR, risk ratio; HR, hazard ratio; LR, likelihood ratio; AUROC, area under the receiver operating characteristic; SD, standard deviation.
