# Supplementary material for: Predicting in-hospital mortality in children in low- and middle-income countries: A systematic review and meta-analysis of vital signs and anthropometric measurements
Source: PLoS One. 2025 Nov 10;20(11):e0336233. doi: 10.1371/journal.pone.0336233 (PMC12599941; doi:10.1371/journal.pone.0336233)
Supplement: S1 Fig — (PDF) [file pone.0336233.s004.pdf]

S1 Fig. Forest plots of abnormal respiratory parameters compared to control on in-hospital mortality

a. Hypoxaemia

Study or Subgroup	Hypoxaemia Events	Hypoxaemia Total	No hypoxaemia Events	No hypoxaemia Total	Weight	Odds Ratio M-H, Random, 95% CI
**1.1.1 Pneumonia**						
Abdulkadir et al 2015	17	83	0	117	0.8%	61.84 [3.66, 1044.98]
Awasthi et al 2023	45	980	48	5765	3.7%	5.73 [3.79, 8.66]
Dembele et al 2019	71	565	127	3740	3.8%	4.09 [3.01, 5.55]
Demers et al 2000	15	109	11	217	3.0%	2.99 [1.32, 6.75]
Eckerle et al 2022	3	79	18	805	2.2%	1.73 [0.50, 5.99]
Gallagher et al 2023	42	536	34	1574	3.6%	3.85 [2.42, 6.12]
Kapoor et al 2022	15	65	2	115	1.8%	16.95 [3.74, 76.92]
Kuti et al 2013	6	73	9	316	2.5%	3.05 [1.05, 8.87]
Macpherson et al 2019	25	179	35	631	3.5%	2.76 [1.61, 4.76]
Nantanda et al 2008	18	62	6	95	2.7%	6.07 [2.25, 16.36]
Odeyemi et al 2021	12	49	1	80	1.2%	25.62 [3.21, 204.48]
Rahman et al 2021	13	77	2	90	1.8%	8.94 [1.95, 40.99]
Ramakrishna et al 2012	19	87	6	146	2.7%	6.52 [2.49, 17.07]
Sigauque et al 2009	18	177	20	407	3.3%	2.19 [1.13, 4.25]
Subtotal (95% CI)		3121		14098	36.8%	4.22 [3.20, 5.58]
Total events	319		319			
Heterogeneity: $Tau^2 = 0.10$; $Chi^2 = 23.64$, df = 13 (P = 0.03); $I^2 = 45\%$						
Test for overall effect: Z = 10.14 (P < 0.00001)						
**1.1.2 Diarrhoea**						
Alege et al 2024	31	38	27	208	2.8%	29.69 [11.90, 74.08]
Chisti et al 2011-1	22	108	2	90	1.9%	11.26 [2.57, 49.33]
Chisti et al 2012	25	119	4	139	2.5%	8.98 [3.02, 26.64]
Shahunja et al 2013	19	71	4	80	2.4%	6.94 [2.23, 21.59]
Shahunja et al 2020	29	68	16	333	3.2%	14.73 [7.35, 29.52]
Talbert et al 2019	25	75	96	2551	3.5%	12.79 [7.59, 21.54]
Subtotal (95% CI)		479		3401	16.3%	13.47 [9.65, 18.79]
Total events	151		149			
Heterogeneity: $Tau^2 = 0.00$; $Chi^2 = 4.93$, df = 5 (P = 0.42); $I^2 = 0\%$						
Test for overall effect: Z = 15.31 (P < 0.00001)						
**1.1.3 Malnutrition**						
Kassaw et al 2021	40	182	14	290	3.3%	5.55 [2.92, 10.55]
Kintwa et al 2021	12	29	7	111	2.5%	10.49 [3.62, 30.39]
Maitland et al 2006	21	137	31	475	3.4%	2.59 [1.44, 4.68]
Nakubeera-Barungi et al 2018	6	25	33	375	2.7%	3.27 [1.22, 8.76]
Talbert et al 2012	22	64	100	528	3.5%	2.24 [1.28, 3.92]
Subtotal (95% CI)		437		1779	15.4%	3.73 [2.26, 6.15]
Total events	101		185			
Heterogeneity: $Tau^2 = 0.18$; $Chi^2 = 9.52$, df = 4 (P = 0.05); $I^2 = 58\%$						
Test for overall effect: Z = 5.15 (P < 0.00001)						
**1.1.4 Other**						
Adegoke et al 2012	7	25	22	286	2.7%	4.67 [1.76, 12.38]
Airlangga et al 2024	5	27	4	249	2.0%	13.92 [3.48, 55.62]
Bains et al 2012	46	52	81	725	2.9%	60.95 [25.24, 147.18]
Ekoube et al 2024	13	127	10	88	2.9%	0.89 [0.37, 2.13]
Gupta et al 2023	44	146	15	254	3.3%	6.87 [3.66, 12.91]
Kumar et al 2003	26	168	18	931	3.3%	9.29 [4.96, 17.38]
Muhanuzi et al 2019	36	79	15	86	3.2%	3.96 [1.95, 8.07]
Olupot-Olupot et al 2020	14	49	49	613	3.2%	4.60 [2.32, 9.13]
Orimadegun et al 2014	19	110	11	259	3.1%	4.71 [2.16, 10.27]
Shamsi et al 2023	6	92	8	42	2.4%	0.30 [0.10, 0.92]
Sharma et al 2021	13	19	14	81	2.4%	10.37 [3.36, 31.97]
Subtotal (95% CI)		894		3614	31.5%	5.11 [2.53, 10.34]
Total events	229		247			
Heterogeneity: $Tau^2 = 1.21$; $Chi^2 = 78.48$, df = 10 (P < 0.00001); $I^2 = 87\%$						
Test for overall effect: Z = 4.54 (P < 0.00001)						
Total (95% CI)		4931		22892	100.0%	5.53 [4.18, 7.30]
Total events	800		900			
Heterogeneity: $Tau^2 = 0.50$; $Chi^2 = 161.04$, df = 35 (P < 0.00001); $I^2 = 78\%$						
Test for overall effect: Z = 12.02 (P < 0.00001)						
Test for subgroup differences: $Chi^2 = 32.39$, df = 3 (P < 0.00001), $I^2 = 90.7\%$						

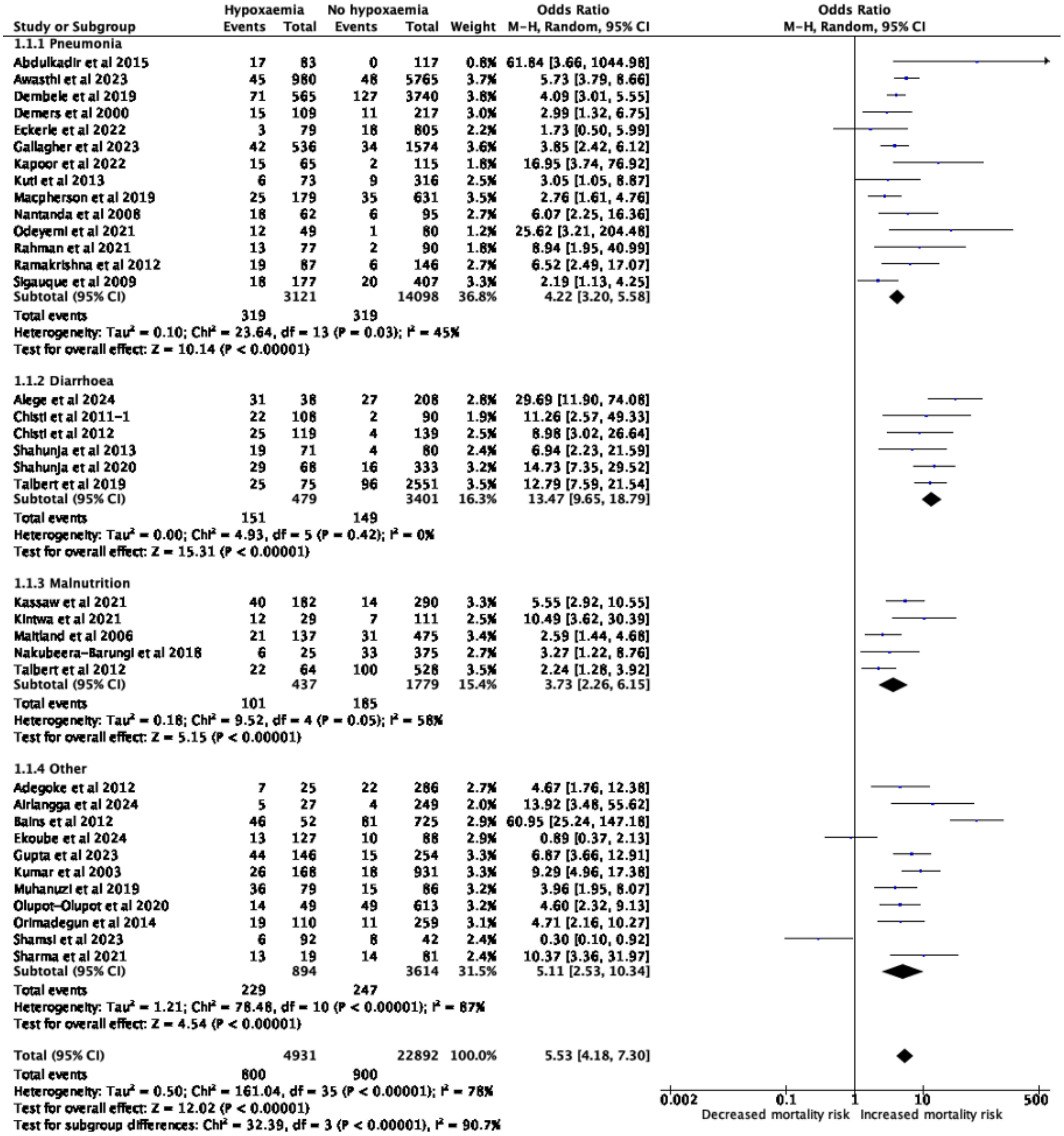

b. Hypoxaemia (cut-off values)

Study or Subgroup	Hypoxaemia Events	Hypoxaemia Total	No hypoxaemia Events	No hypoxaemia Total	Weight	Odds Ratio M-H, Random, 95% CI
13.1.1 <95% Pneumonia						
Eckerle et al 2022	3	79	18	805	2.3%	1.73 [0.50, 5.99]
Nantanda et al 2008	18	62	6	95	2.7%	6.07 [2.25, 16.36]
Subtotal (95% CI)		141		900	5.0%	3.43 [1.00, 11.78]
Total events	21		24			
Heterogeneity: Tau² = 0.47; Chi² = 2.43, df = 1 (P = 0.12); I² = 59%						
Test for overall effect: Z = 1.96 (P = 0.05)						
13.1.2 <95% Malnutrition						
Maitland et al 2006	21	137	31	475	3.5%	2.59 [1.44, 4.68]
Nakubeera–Barungi et al 2018	6	25	33	375	2.7%	3.27 [1.22, 8.76]
Talbert et al 2012	22	64	100	528	3.6%	2.24 [1.28, 3.92]
Subtotal (95% CI)		226		1378	9.9%	2.51 [1.73, 3.66]
Total events	49		164			
Heterogeneity: Tau² = 0.00; Chi² = 0.45, df = 2 (P = 0.80); I² = 0%						
Test for overall effect: Z = 4.81 (P < 0.00001)						
13.1.3 <95% Other						
Airlangga et al 2024 (1)	5	27	4	249	2.0%	13.92 [3.48, 55.62]
Ekoube et al 2024 (2)	13	127	10	88	3.0%	0.89 [0.37, 2.13]
Muhanuzi et al 2019	36	79	15	86	3.3%	3.96 [1.95, 8.07]
Olupot–Olupot et al 2020	14	49	49	613	3.4%	4.60 [2.32, 9.13]
Shamsi et al 2023 (3)	6	92	8	42	2.5%	0.30 [0.10, 0.92]
Sharma et al 2021	13	19	14	81	2.5%	10.37 [3.36, 31.97]
Subtotal (95% CI)		393		1159	16.6%	2.91 [1.06, 7.98]
Total events	87		100			
Heterogeneity: Tau² = 1.33; Chi² = 35.46, df = 5 (P < 0.00001); I² = 86%						
Test for overall effect: Z = 2.07 (P = 0.04)						
13.1.4 <90% Pneumonia						
Abdulkadir et al 2015	17	83	0	117	0.8%	61.84 [3.66, 1044.98]
Awasthi et al 2023	45	980	48	5765	3.9%	5.73 [3.79, 8.66]
Dembele et al 2019	71	565	127	3740	4.0%	4.09 [3.01, 5.55]
Demers et al 2000	15	109	11	217	3.1%	2.99 [1.32, 6.75]
Gallagher et al 2023	42	536	34	1574	3.8%	3.85 [2.42, 6.12]
Kapoor et al 2022	15	65	2	115	1.8%	16.95 [3.74, 76.92]
Kuti et al 2013	6	73	9	316	2.6%	3.05 [1.05, 8.87]
Macpherson et al 2019	25	179	35	631	3.6%	2.76 [1.61, 4.76]
Odeyemi et al 2021	12	49	1	80	1.2%	25.62 [3.21, 204.48]
Rahman et al 2021	13	77	2	90	1.8%	8.94 [1.95, 40.99]
Ramakrishna et al 2012	19	87	6	146	2.8%	6.52 [2.49, 17.07]
Sigauque et al 2009	18	177	20	407	3.4%	2.19 [1.13, 4.25]
Subtotal (95% CI)		2980		13198	32.8%	4.30 [3.20, 5.78]
Total events	298		295			
Heterogeneity: Tau² = 0.10; Chi² = 21.16, df = 11 (P = 0.03); I² = 48%						
Test for overall effect: Z = 9.69 (P < 0.00001)						
13.1.5 <90% Diarrhoea						
Chisti et al 2011–1	22	108	2	90	1.9%	11.26 [2.57, 49.33]
Chisti et al 2012	25	119	4	139	2.5%	8.98 [3.02, 26.64]
Shahunja et al 2013	19	71	4	80	2.5%	6.94 [2.23, 21.59]
Shahunja et al 2020	29	68	16	333	3.3%	14.73 [7.35, 29.52]
Talbert et al 2019	25	75	96	2551	3.7%	12.79 [7.59, 21.54]
Subtotal (95% CI)		441		3193	13.9%	11.94 [8.35, 17.06]
Total events	120		122			
Heterogeneity: Tau² = 0.00; Chi² = 1.72, df = 4 (P = 0.79); I² = 0%						
Test for overall effect: Z = 13.59 (P < 0.00001)						
13.1.6 <90% Malnutrition						
Kassaw et al 2021	40	182	14	290	3.4%	5.55 [2.92, 10.55]
Kintwa et al 2021	12	29	7	111	2.6%	10.49 [3.62, 30.39]
Subtotal (95% CI)		211		401	6.0%	6.60 [3.78, 11.51]
Total events	52		21			
Heterogeneity: Tau² = 0.00; Chi² = 1.02, df = 1 (P = 0.31); I² = 2%						
Test for overall effect: Z = 6.64 (P < 0.00001)						
13.1.7 <90% Other						
Adegoke et al 2012	7	25	22	286	2.8%	4.67 [1.76, 12.38]
Bains et al 2012	46	52	81	725	2.9%	60.95 [25.24, 147.18]
Gupta et al 2023	44	146	15	254	3.5%	6.87 [3.66, 12.91]
Kumar et al 2003	26	168	18	931	3.5%	9.29 [4.96, 17.38]
Orimadegun et al 2014	19	110	11	259	3.2%	4.71 [2.16, 10.27]
Subtotal (95% CI)		501		2455	15.8%	9.60 [4.23, 21.81]
Total events	142		147			
Heterogeneity: Tau² = 0.72; Chi² = 23.34, df = 4 (P = 0.0001); I² = 83%						
Test for overall effect: Z = 5.40 (P < 0.00001)						
Total (95% CI)		4893		22684	100.0%	5.25 [3.99, 6.89]
Total events	769		873			
Heterogeneity: Tau² = 0.46; Chi² = 146.18, df = 34 (P < 0.00001); I² = 77%						
Test for overall effect: Z = 11.91 (P < 0.00001)						
Test for subgroup differences: Chi² = 41.31, df = 6 (P < 0.00001), I² = 85.5%						

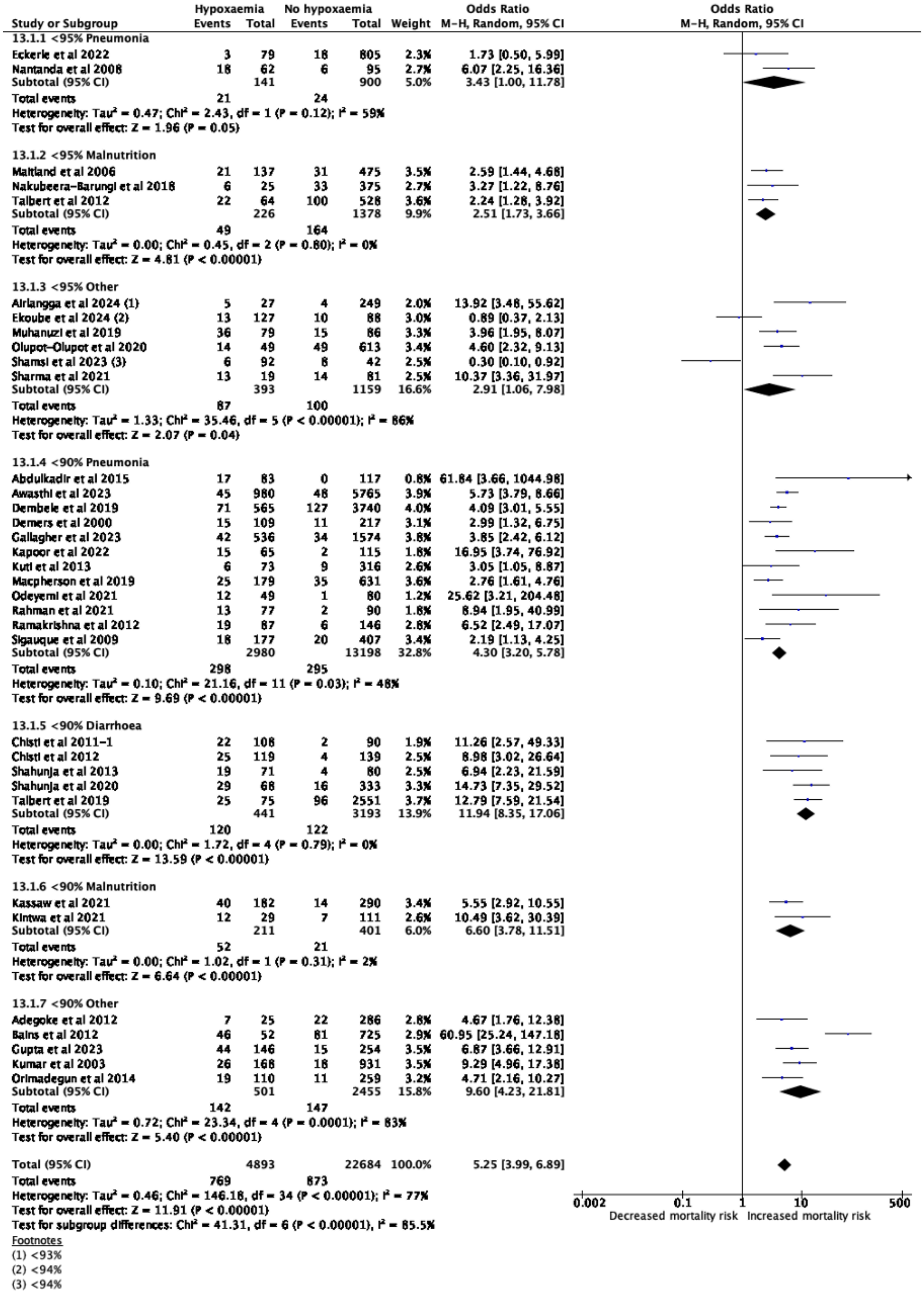

Footnotes
(1) <93%
(2) <94%
(3) <94%

c. Tachypnoea

Study or Subgroup	Tachypnoea Events	Tachypnoea Total	No tachypnoea Events	No tachypnoea Total	Weight	Odds Ratio M-H, Random, 95% CI
**2.1.1 Pneumonia**						
Agweyu et al 2018	394	9657	125	1254	5.0%	0.38 [0.31, 0.47]
Awasthi et al 2023	86	5305	7	1440	4.1%	3.37 [1.56, 7.30]
Dembele et al 2019	337	3597	16	679	4.6%	4.28 [2.58, 7.12]
Demers et al 2000	25	152	24	243	4.4%	1.80 [0.98, 3.28]
Eckerle et al 2022	18	738	3	146	3.1%	1.19 [0.35, 4.10]
Lazzerini et al 2016	5139	80813	364	4887	5.1%	0.84 [0.76, 0.94]
Macpherson et al 2019	94	1126	17	376	4.6%	1.92 [1.13, 3.27]
Shann et al 1989	11	86	12	92	3.9%	0.98 [0.41, 2.35]
Sharma et al 2021	15	47	12	53	3.9%	1.60 [0.66, 3.90]
Spooner et al 1989	37	353	22	235	4.5%	1.13 [0.65, 1.98]
Subtotal (95% CI)		101874		9405	43.2%	1.39 [0.86, 2.25]
Total events	6156		602			
Heterogeneity: Tau² = 0.49; Chi² = 128.41, df = 9 (P < 0.00001); I² = 93%						
Test for overall effect: Z = 1.34 (P = 0.18)						
**2.1.2 Malnutrition**						
Jarso et al 2015	53	497	35	328	4.7%	1.00 [0.64, 1.57]
Kambale et al 2020	13	214	27	307	4.3%	0.67 [0.34, 1.33]
Kintwa et al 2021	4	9	15	131	2.8%	6.19 [1.49, 25.61]
Maitland et al 2006	31	305	28	498	4.6%	1.90 [1.12, 3.23]
Nakubeera-Barungi et al 2018	11	58	28	342	4.1%	2.62 [1.23, 5.62]
Talbert et al 2012	33	161	89	431	4.7%	0.99 [0.63, 1.55]
Subtotal (95% CI)		1244		2037	25.2%	1.40 [0.89, 2.21]
Total events	145		222			
Heterogeneity: Tau² = 0.21; Chi² = 16.08, df = 5 (P = 0.007); I² = 69%						
Test for overall effect: Z = 1.45 (P = 0.15)						
**2.1.3 General**						
Bains et al 2012	85	196	42	581	4.7%	9.83 [6.44, 14.99]
Berkley et al 2003	113	2289	124	5497	5.0%	2.25 [1.74, 2.92]
Kumar et al 2003	12	202	32	897	4.3%	1.71 [0.86, 3.38]
Mujuru et al 2012	8	72	8	101	3.6%	1.45 [0.52, 4.07]
Subtotal (95% CI)		2759		7076	17.5%	2.85 [1.15, 7.07]
Total events	218		206			
Heterogeneity: Tau² = 0.75; Chi² = 39.52, df = 3 (P < 0.00001); I² = 92%						
Test for overall effect: Z = 2.26 (P = 0.02)						
**2.1.4 Other**						
Ahmed et al 2011	16	34	19	66	3.9%	2.20 [0.93, 5.19]
Mishra et al 2023	8	38	2	12	2.3%	1.33 [0.24, 7.35]
Samuel et al 2014	8	105	4	30	3.1%	0.54 [0.15, 1.92]
Talbert et al 2019	62	768	59	1858	4.8%	2.68 [1.86, 3.86]
Subtotal (95% CI)		945		1966	14.1%	1.78 [0.93, 3.40]
Total events	94		84			
Heterogeneity: Tau² = 0.21; Chi² = 6.07, df = 3 (P = 0.11); I² = 51%						
Test for overall effect: Z = 1.75 (P = 0.08)						
Total (95% CI)		106822		20484	100.0%	1.65 [1.16, 2.34]
Total events	6613		1114			
Heterogeneity: Tau² = 0.64; Chi² = 341.70, df = 23 (P < 0.00001); I² = 93%						
Test for overall effect: Z = 2.76 (P = 0.006)						
Test for subgroup differences: Chi² = 2.29, df = 3 (P = 0.51), I² = 0%						

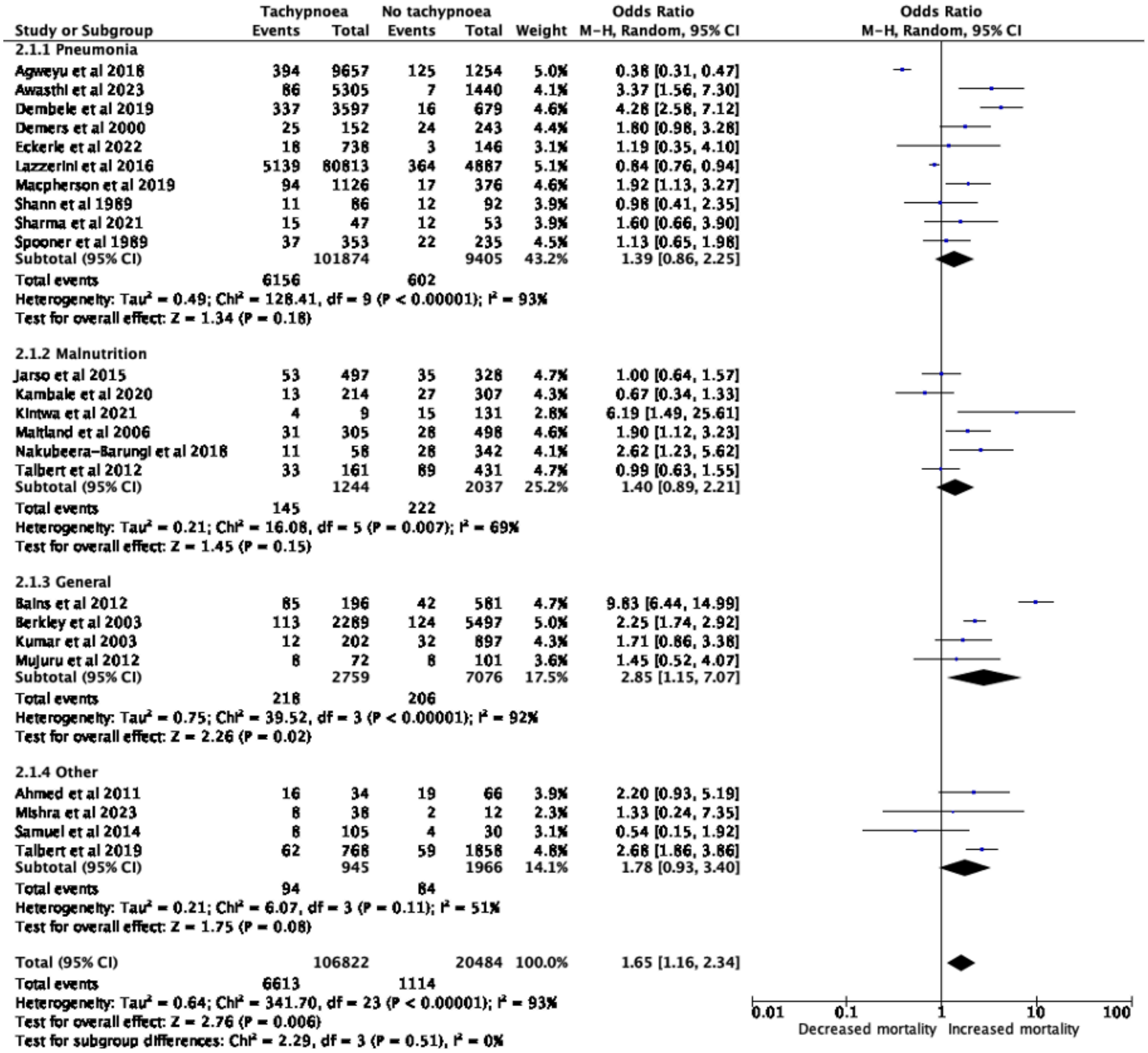

d. Bradypnoea

Study or Subgroup	Bradypnoea Events	Bradypnoea Total	No bradypnoea Events	No bradypnoea Total	Weight	Odds Ratio M-H, Random, 95% CI
**3.1.1 Malnutrition**						
Bashaka et al 2019	1	8	17	125	15.9%	0.91 [0.11, 7.84]
Jarso et al 2015	10	74	78	751	27.4%	1.35 [0.67, 2.73]
Kambale et al 2020	4	8	36	513	21.6%	13.25 [3.18, 55.19]
Subtotal (95% CI)		90		1389	64.8%	2.62 [0.51, 13.34]
Total events	15		131			
Heterogeneity: Tau² = 1.53; Chi² = 8.62, df = 2 (P = 0.01); I² = 77%						
Test for overall effect: Z = 1.16 (P = 0.25)						
**3.1.2 Other**						
Ahmed et al 2011	5	13	30	87	23.5%	1.19 [0.36, 3.95]
Muhanuzi et al 2019	11	11	40	154	11.7%	65.02 [3.75, 1128.65]
Subtotal (95% CI)		24		241	35.2%	7.35 [0.10, 552.06]
Total events	16		70			
Heterogeneity: Tau² = 8.54; Chi² = 7.84, df = 1 (P = 0.005); I² = 87%						
Test for overall effect: Z = 0.91 (P = 0.37)						
Total (95% CI)		114		1630	100.0%	3.17 [0.91, 11.07]
Total events	31		201			
Heterogeneity: Tau² = 1.36; Chi² = 15.30, df = 4 (P = 0.004); I² = 74%						
Test for overall effect: Z = 1.81 (P = 0.07)						
Test for subgroup differences: Chi² = 0.19, df = 1 (P = 0.66), I² = 0%						

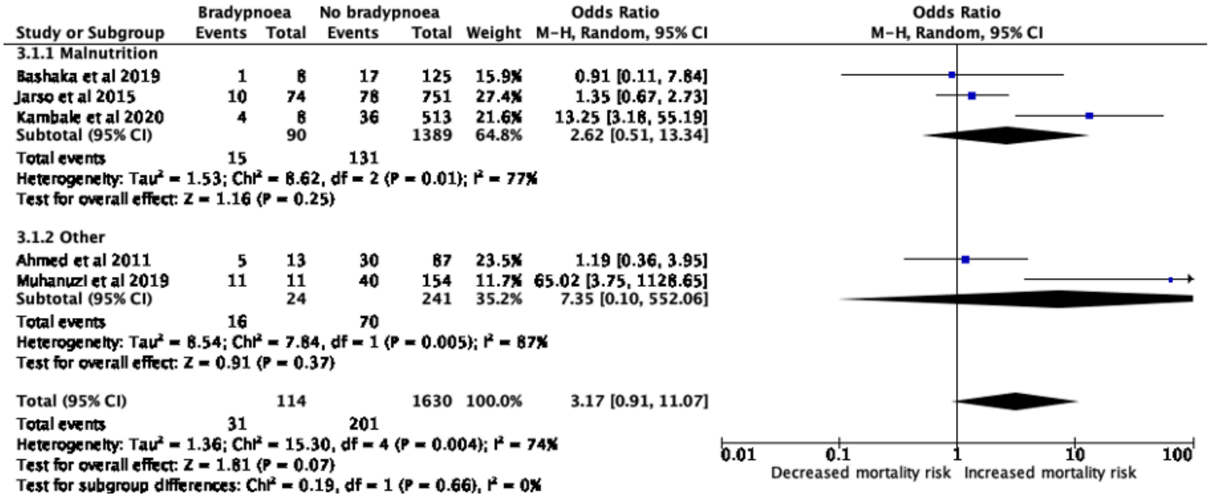

OR, Odds Ratio; CI, Confidence Interval.
