# Supplementary material for: Predicting in-hospital mortality in children in low- and middle-income countries: A systematic review and meta-analysis of vital signs and anthropometric measurements
Source: PLoS One. 2025 Nov 10;20(11):e0336233. doi: 10.1371/journal.pone.0336233 (PMC12599941; doi:10.1371/journal.pone.0336233)
Supplement: S2 Fig — (PDF) [file pone.0336233.s005.pdf]

S2 Fig. Forest plots of abnormal circulatory parameters compared to control on in-hospital mortality

a. Tachycardia

Study or Subgroup	Tachycardia Events	Tachycardia Total	No tachycardia Events	No tachycardia Total	Weight	Odds Ratio M-H, Random, 95% CI
Ahmed et al 2011	10	33	25	67	7.7%	0.73 [0.30, 1.78]
Alam et al 2023	15	54	3	44	5.3%	5.26 [1.41, 19.58]
Bains et al 2012	44	126	83	651	11.2%	3.67 [2.38, 5.66]
Bashaka et al 2019	10	34	8	100	6.8%	4.79 [1.71, 13.46]
Dembele et al 2019	69	1442	125	2816	12.0%	1.08 [0.80, 1.46]
Eckerle et al 2022	4	169	1	185	2.5%	4.46 [0.49, 40.31]
Ekoube et al 2024	18	163	5	78	6.8%	1.81 [0.65, 5.08]
Fouad et al 2011	9	10	41	90	2.7%	10.76 [1.31, 88.47]
Jarso et al 2015	5	78	83	747	7.4%	0.55 [0.22, 1.39]
Kumar et al 2003	17	291	27	808	9.8%	1.79 [0.96, 3.34]
Mishra et al 2023	10	46	0	4	1.5%	2.59 [0.13, 52.07]
Muoneke et al 2012	17	94	2	46	4.4%	4.86 [1.07, 22.01]
Spooner et al 1989	21	216	38	372	10.2%	0.95 [0.54, 1.66]
Talbert et al 2019	43	1049	78	2394	11.5%	1.27 [0.87, 1.86]
Total (95% CI)		3805		8402	100.0%	1.80 [1.22, 2.66]
Total events	292		519			

Heterogeneity: Tau² = 0.31; Chi² = 46.81, df = 13 (P < 0.0001); I² = 72%
Test for overall effect: Z = 2.94 (P = 0.003)

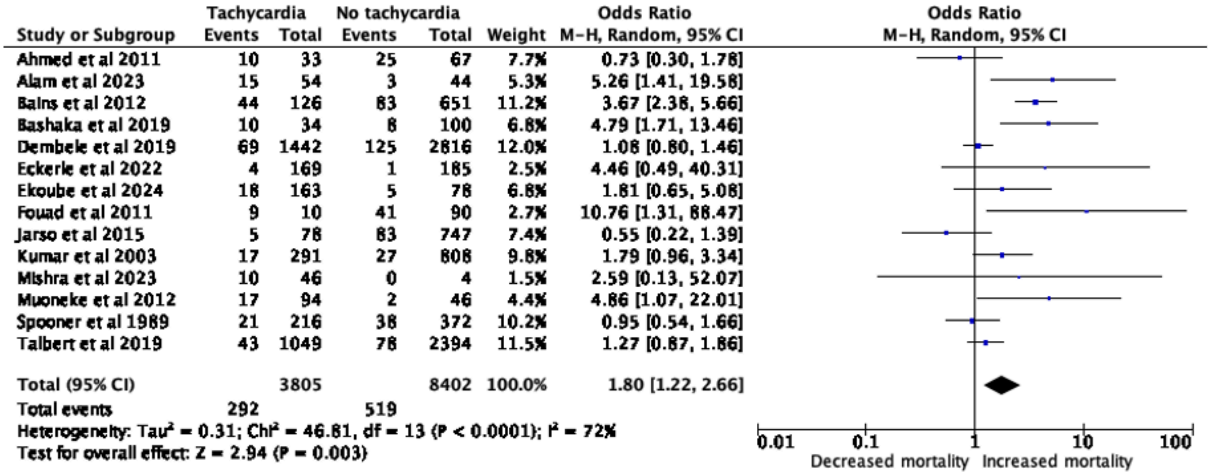

b. Bradycardia

Study or Subgroup	Bradycardia Events	Bradycardia Total	No bradycardia Events	No bradycardia Total	Weight	Odds Ratio M-H, Random, 95% CI
Ahmed et al 2011	21	47	14	53	17.2%	2.25 [0.97, 5.21]
Bokade et al 2014	17	49	17	127	17.6%	3.44 [1.58, 7.49]
Broek, van den et al 2005	26	29	74	171	14.5%	11.36 [3.31, 38.97]
Fouad et al 2011	33	74	17	26	16.6%	0.43 [0.17, 1.08]
Jarso et al 2015	22	100	66	725	19.0%	2.82 [1.65, 4.82]
Maitland et al 2006	7	13	52	790	15.2%	16.56 [5.37, 51.06]
Total (95% CI)		312		1892	100.0%	3.29 [1.38, 7.83]
Total events	126		240			

Heterogeneity: Tau² = 0.96; Chi² = 30.90, df = 5 (P < 0.00001); I² = 84%
Test for overall effect: Z = 2.69 (P = 0.007)

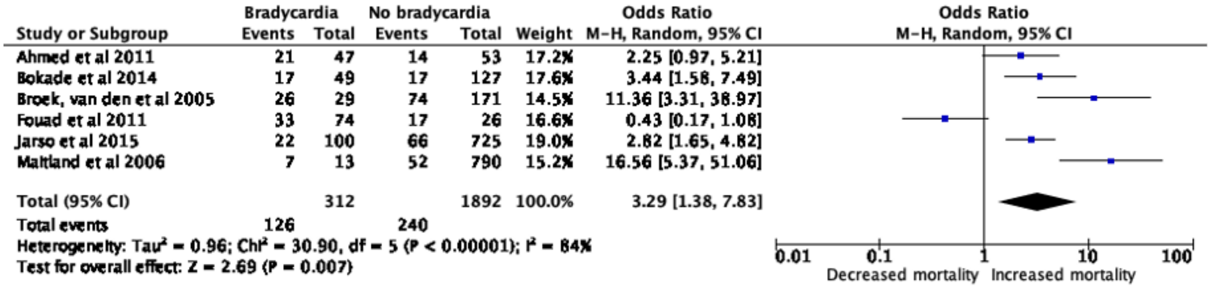

c. Hypertension

Study or Subgroup	Hypertension Events	Hypertension Total	No hypertension Events	No hypertension Total	Weight	Odds Ratio M-H, Random, 95% CI
Ahmed Ali et al 2017	2	21	3	18	16.5%	0.53 [0.08, 3.56]
Ahmed et al 2011	4	10	28	87	32.8%	1.40 [0.37, 5.38]
Alao et al 2023	3	32	22	83	35.7%	0.29 [0.08, 1.04]
Fouad et al 2011	2	4	48	96	15.1%	1.00 [0.14, 7.39]
Total (95% CI)		67		284	100.0%	0.64 [0.29, 1.41]
Total events	11		101			

Heterogeneity: Tau² = 0.02; Chi² = 3.09, df = 3 (P = 0.38); I² = 3%
Test for overall effect: Z = 1.10 (P = 0.27)

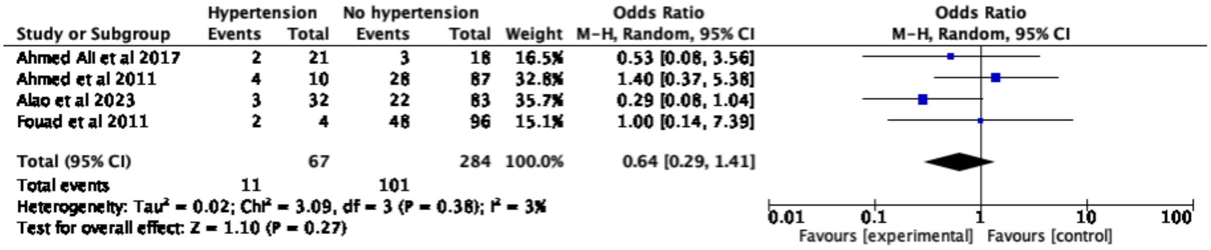

d. Hypotension

Study or Subgroup	Hypotension Events	Hypotension Total	No hypotension Events	No hypotension Total	Weight	Odds Ratio M-H, Random, 95% CI
Abdel Baseer et al 2022	23	33	11	70	12.9%	12.34 [4.62, 32.96]
Abrar et al 2016	9	29	2	33	7.5%	6.97 [1.36, 35.67]
Adegoke et al 2012	9	34	20	277	13.9%	4.63 [1.90, 11.24]
Ahmed et al 2011	19	45	13	52	14.2%	2.19 [0.93, 5.19]
Alam et al 2023	12	38	6	60	11.8%	4.15 [1.40, 12.30]
Alao et al 2023	1	2	24	113	3.3%	3.71 [0.22, 61.49]
Bashaka et al 2019	3	8	5	13	6.5%	0.96 [0.16, 5.90]
Fouad et al 2011	29	47	21	53	14.9%	2.46 [1.10, 5.49]
Kumar et al 2003	6	16	38	1083	12.1%	16.50 [5.70, 47.75]
Mishra et al 2023	10	48	0	2	2.8%	1.36 [0.06, 30.64]
Total (95% CI)		300		1756	100.0%	4.42 [2.54, 7.70]
Total events	121		140			

Heterogeneity: Tau² = 0.37; Chi² = 18.59, df = 9 (P = 0.03); I² = 52%
Test for overall effect: Z = 5.24 (P < 0.00001)

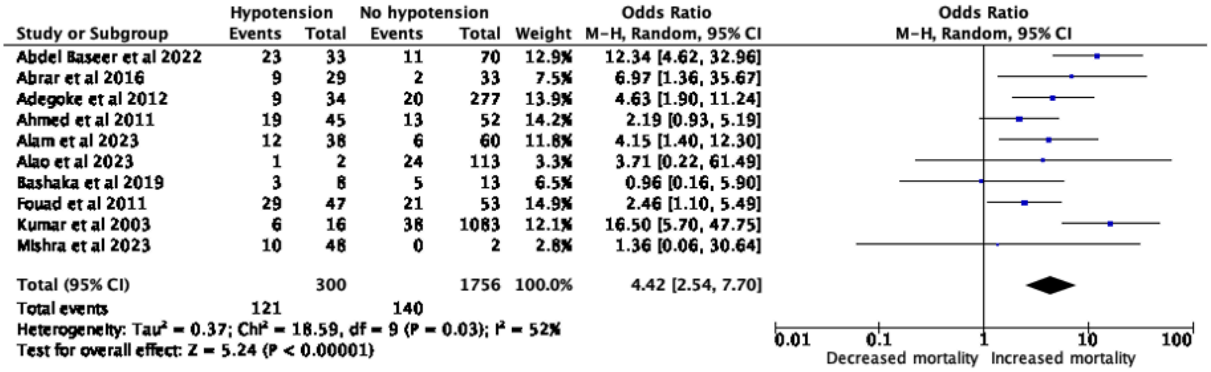

OR, Odds Ratio; CI, Confidence Interval.
