# Supplementary material for: Predicting in-hospital mortality in children in low- and middle-income countries: A systematic review and meta-analysis of vital signs and anthropometric measurements
Source: PLoS One. 2025 Nov 10;20(11):e0336233. doi: 10.1371/journal.pone.0336233 (PMC12599941; doi:10.1371/journal.pone.0336233)
Supplement: S3 Fig — (PDF) [file pone.0336233.s006.pdf]

S3 Fig. Forest plots of abnormal temperature compared to control on in-hospital mortality

a. Hyperthermia

Study or Subgroup	Hyperthermia Events	Hyperthermia Total	No hyperthermia Events	No hyperthermia Total	Weight	Odds Ratio M-H, Random, 95% CI
8.1.1 Pneumonia						
Agweyu et al 2018	158	2026	394	9587	3.8%	1.97 [1.63, 2.39]
Dembele et al 2019	72	934	126	3371	3.7%	2.15 [1.59, 2.90]
Lazzerini et al 2016	1699	28804	3400	51349	3.9%	0.88 [0.83, 0.94]
Macpherson et al 2019	47	667	72	966	3.6%	0.94 [0.64, 1.38]
Odeyemi et al 2021	10	43	3	86	1.7%	8.38 [2.17, 32.40]
Sharma et al 2021	19	80	8	20	2.2%	0.47 [0.17, 1.31]
Sigauque et al 2009	158	530	3	240	2.0%	33.55 [10.58, 106.38]
Subtotal (95% CI)		33084		65619	20.9%	1.95 [1.14, 3.32]
Total events	2163		4006			
Heterogeneity: $Tau^2 = 0.40$; $Chi^2 = 137.43$, df = 6 ($P < 0.00001$); $I^2 = 96\%$						
Test for overall effect: Z = 2.45 (P = 0.01)						
8.1.2 Diarrhoea						
Alege et al 2024	11	42	47	204	2.8%	1.19 [0.55, 2.54]
Chisti et al 2011-1	4	64	25	169	2.1%	0.38 [0.13, 1.15]
Lindtjorn et al 1991	13	32	8	52	2.2%	3.76 [1.34, 10.56]
Shahunja et al 2013	19	130	4	21	2.0%	0.73 [0.22, 2.40]
Shahunja et al 2020	28	293	17	108	3.0%	0.57 [0.30, 1.08]
Subtotal (95% CI)		561		554	12.1%	0.93 [0.45, 1.89]
Total events	75		101			
Heterogeneity: $Tau^2 = 0.44$; $Chi^2 = 12.28$, df = 4 ($P = 0.02$); $I^2 = 67\%$						
Test for overall effect: Z = 0.21 (P = 0.84)						
8.1.3 Malnutrition						
Bashaka et al 2019	5	28	13	107	2.1%	1.57 [0.51, 4.85]
Girum et al 2017	39	92	12	453	2.9%	27.04 [13.34, 54.84]
Ikobah et al 2022	6	54	1	34	0.9%	4.13 [0.47, 35.87]
Kambale et al 2020	4	62	30	450	2.1%	0.97 [0.33, 2.84]
Maitland et al 2006	24	363	35	440	3.2%	0.82 [0.48, 1.40]
Sturgeon et al 2023	32	324	29	357	3.3%	1.24 [0.73, 2.10]
Talbert et al 2012	47	229	75	363	3.5%	0.99 [0.66, 1.49]
Subtotal (95% CI)		1152		2204	18.0%	2.00 [0.78, 5.15]
Total events	157		195			
Heterogeneity: $Tau^2 = 1.39$; $Chi^2 = 75.04$, df = 6 ($P < 0.00001$); $I^2 = 92\%$						
Test for overall effect: Z = 1.43 (P = 0.15)						
8.1.4 Malaria						
Ilunga-Ilunga et al 2015	18	139	62	1211	3.2%	2.76 [1.58, 4.81]
Kouéta et al 2007	22	42	50	102	2.9%	1.14 [0.56, 2.35]
Olupot-Olupot et al 2020	31	411	32	251	3.3%	0.56 [0.33, 0.94]
Subtotal (95% CI)		592		1564	9.4%	1.20 [0.44, 3.27]
Total events	71		144			
Heterogeneity: $Tau^2 = 0.69$; $Chi^2 = 17.02$, df = 2 ($P = 0.0002$); $I^2 = 88\%$						
Test for overall effect: Z = 0.37 (P = 0.71)						
8.1.5 General						
Berkley et al 2003	54	2544	169	5103	3.7%	0.63 [0.46, 0.86]
Jofiro et al 2018	15	48	323	0		Not estimable
Mujuru et al 2012	26	326	28	411	3.2%	1.19 [0.68, 2.06]
Tette et al 2016	83	162	32	73	3.2%	1.35 [0.77, 2.35]
Subtotal (95% CI)		3080		5587	10.1%	0.96 [0.57, 1.62]
Total events	178		552			
Heterogeneity: $Tau^2 = 0.15$; $Chi^2 = 7.43$, df = 2 ($P = 0.02$); $I^2 = 73\%$						
Test for overall effect: Z = 0.15 (P = 0.88)						
8.1.6 Other						
Ahmed et al 2011	10	39	25	61	2.5%	0.50 [0.21, 1.20]
Airlangga et al 2024	7	232	2	52	1.4%	0.78 [0.16, 3.86]
Alao et al 2023	21	123	4	42	2.1%	1.96 [0.63, 6.07]
Ekoube et al 2024	15	113	8	102	2.5%	1.80 [0.73, 4.44]
Fattahi et al 2022	10	326	6	319	2.2%	1.65 [0.59, 4.60]
Fouad et al 2011	9	37	41	63	2.5%	0.17 [0.07, 0.43]
Mishra et al 2023	8	38	2	12	1.3%	1.33 [0.24, 7.35]
Muhanuzi et al 2019	24	89	27	76	3.0%	0.67 [0.35, 1.30]
Nasir et al 2011	10	65	4	78	1.9%	3.36 [1.00, 11.29]
Nguyen et al 2022	15	86	3	10	1.6%	0.49 [0.11, 2.13]
Shafaei et al 2023	18	122	6	69	2.3%	1.82 [0.69, 4.82]
Shah et al 2020	40	63	10	26	2.4%	2.78 [1.08, 7.14]
Sharma et al 2022	19	80	8	20	2.2%	0.47 [0.17, 1.31]
Talabi et al 2014	5	18	4	27	1.5%	2.21 [0.50, 9.72]
Subtotal (95% CI)		1431		957	29.5%	1.05 [0.65, 1.69]
Total events	211		150			
Heterogeneity: $Tau^2 = 0.50$; $Chi^2 = 36.07$, df = 13 ($P = 0.0006$); $I^2 = 64\%$						
Test for overall effect: Z = 0.21 (P = 0.84)						
Total (95% CI)		39900		76485	100.0%	1.31 [1.04, 1.66]
Total events	2855		5148			
Heterogeneity: $Tau^2 = 0.37$; $Chi^2 = 300.85$, df = 38 ($P < 0.00001$); $I^2 = 87\%$						
Test for overall effect: Z = 2.28 (P = 0.02)						
Test for subgroup differences: $Chi^2 = 5.76$, df = 5 ($P = 0.33$), $I^2 = 13.2\%$						

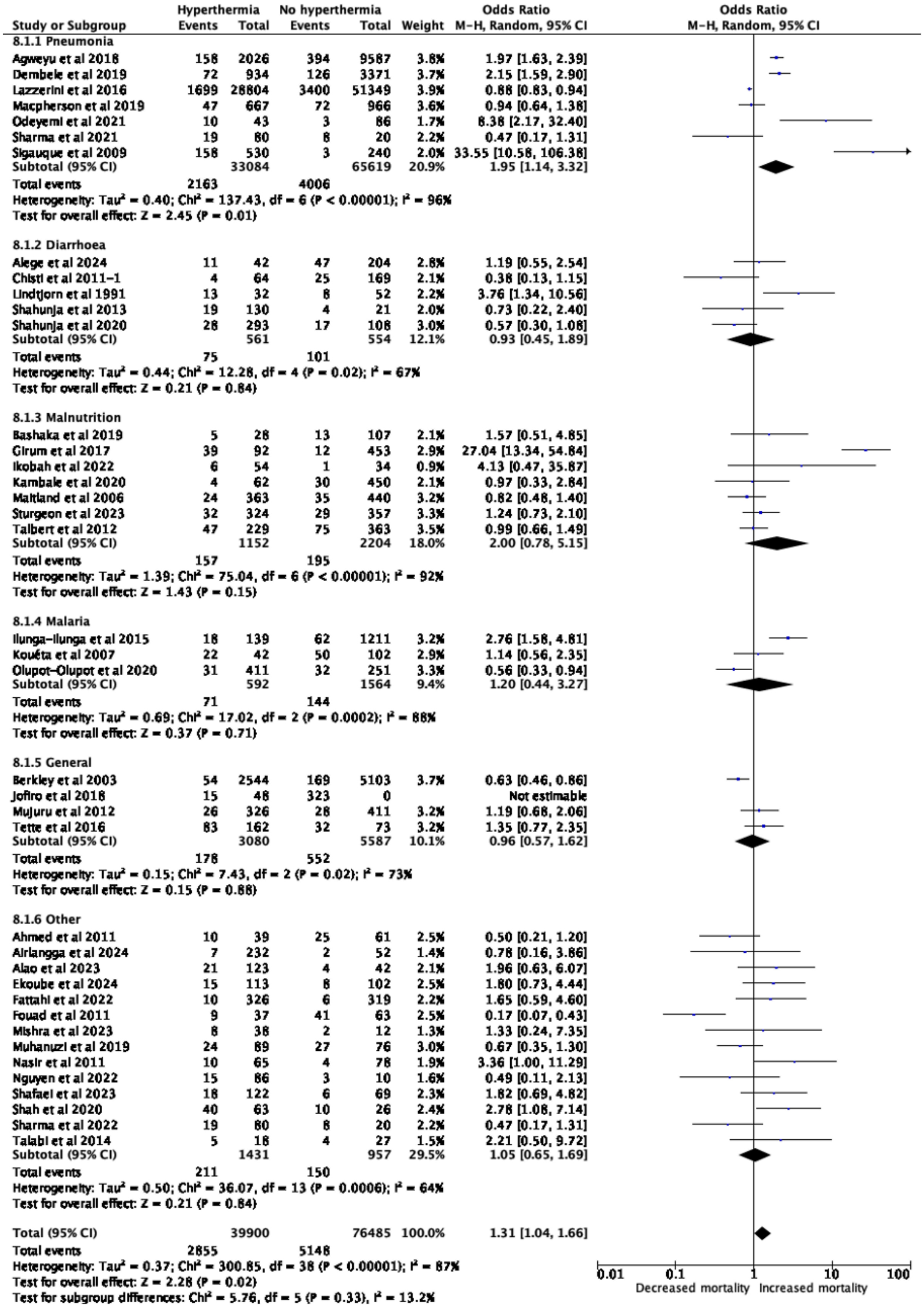

b. Hypothermia

Study or Subgroup	Hypothermia Events	Hypothermia Total	No hypothermia Events	No hypothermia Total	Weight	Odds Ratio M-H, Random, 95% CI
**9.1.1 Malnutrition**						
Jarso et al 2015	7	17	81	808	6.7%	6.28 [2.33, 16.96]
Kambale et al 2020	6	20	34	512	6.5%	6.03 [2.18, 16.67]
Maitland et al 2006	2	11	57	792	3.8%	2.87 [0.60, 13.58]
Roy et al 2013	40	52	63	154	8.9%	4.81 [2.34, 9.90]
Shahunja et al 2013	2	7	21	144	3.3%	2.34 [0.43, 12.87]
Sturgeon et al 2023	9	47	52	634	8.4%	2.65 [1.22, 5.78]
Talbert et al 2009	4	12	121	655	5.3%	2.21 [0.65, 7.45]
Subtotal (95% CI)		166		3699	42.9%	3.89 [2.67, 5.68]
Total events	70		429			
Heterogeneity: $Tau^2 = 0.00$; $Chi^2 = 4.19$, df = 6 ($P = 0.65$); $I^2 = 0\%$						
Test for overall effect: $Z = 7.04$ ($P < 0.00001$)						
**9.1.2 Other**						
Abdel Baseer et al 2022	18	23	16	80	5.8%	14.40 [4.64, 44.67]
Ahmed et al 2011	19	37	16	63	7.7%	3.10 [1.31, 7.32]
Berkley et al 2003	20	223	169	5103	11.3%	2.88 [1.77, 4.67]
Broek, van den et al 2005	24	28	76	172	6.0%	7.58 [2.52, 22.78]
Chisti et al 2011-1	3	15	26	243	4.7%	2.09 [0.55, 7.88]
Fouad et al 2011	8	9	42	91	2.3%	9.33 [1.12, 77.70]
Kuti et al 2013	5	14	10	375	5.0%	20.28 [5.75, 71.55]
Muoneke et al 2012	2	8	17	132	3.4%	2.25 [0.42, 12.09]
Ochora et al 2024	11	85	5	123	6.0%	3.51 [1.17, 10.50]
Shamsi et al 2023	4	59	7	62	4.9%	0.57 [0.16, 2.06]
Subtotal (95% CI)		501		6444	57.1%	4.09 [2.28, 7.33]
Total events	114		384			
Heterogeneity: $Tau^2 = 0.52$; $Chi^2 = 25.28$, df = 9 ($P = 0.003$); $I^2 = 64\%$						
Test for overall effect: $Z = 4.73$ ($P < 0.00001$)						
Total (95% CI)		667		10143	100.0%	3.92 [2.76, 5.58]
Total events	184		813			
Heterogeneity: $Tau^2 = 0.23$; $Chi^2 = 29.52$, df = 16 ($P = 0.02$); $I^2 = 46\%$						
Test for overall effect: $Z = 7.60$ ($P < 0.00001$)						
Test for subgroup differences: $Chi^2 = 0.02$, df = 1 ($P = 0.89$), $I^2 = 0\%$						

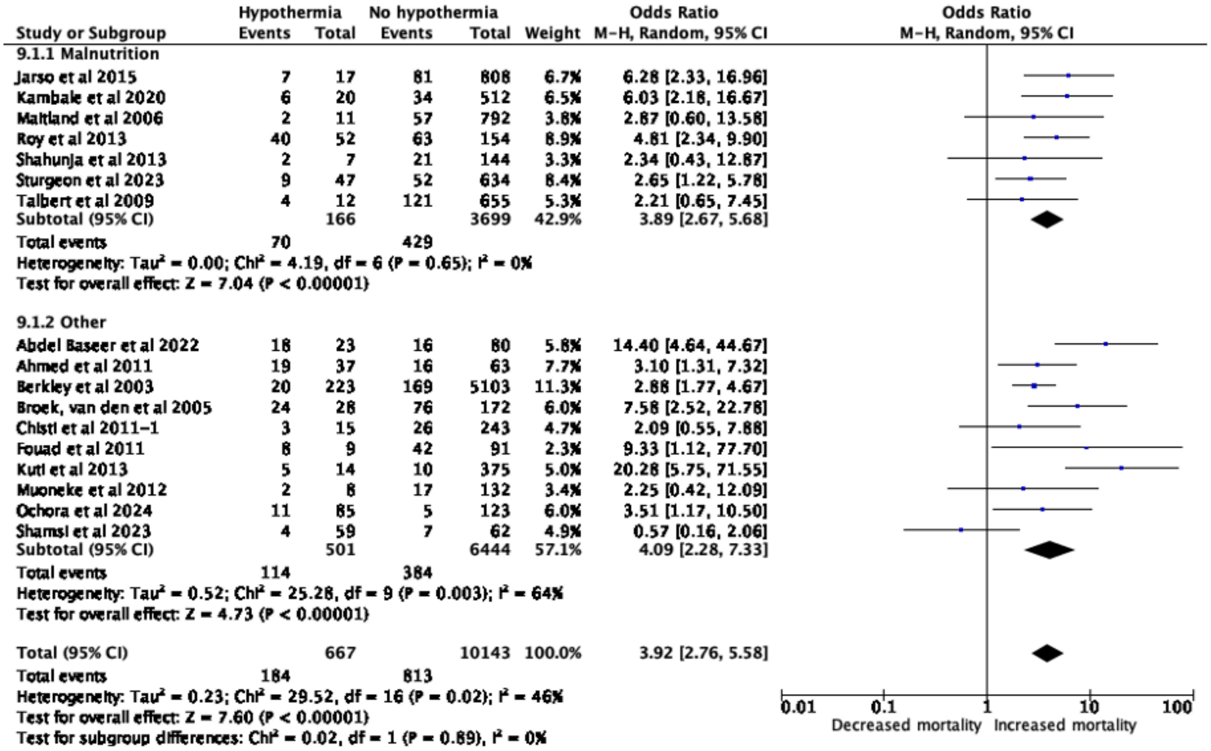

OR, Odds Ratio; CI, Confidence Interval.
