# Supplementary material for: Predicting in-hospital mortality in children in low- and middle-income countries: A systematic review and meta-analysis of vital signs and anthropometric measurements
Source: PLoS One. 2025 Nov 10;20(11):e0336233. doi: 10.1371/journal.pone.0336233 (PMC12599941; doi:10.1371/journal.pone.0336233)
Supplement: S4 Fig — (PDF) [file pone.0336233.s007.pdf]

S4 Fig. Forest plot of abnormal anthropometric measurements compared to control on in-hospital mortality

a. MUAC

	Low MUAC		Normal MUAC			Odds Ratio
Study or Subgroup	Events	Total	Events	Total	Weight	M-H, Random, 95% CI
10.1.1 Malnutrition						
Chiabi et al 2017	21	83	1	23	2.8%	7.45 [0.95, 58.71]
Girum et al 2017	41	277	10	268	6.9%	4.48 [2.20, 9.15]
Jarso et al 2015	60	356	8	260	6.7%	6.39 [3.00, 13.61]
Kambale et al 2020	27	277	10	227	6.7%	2.34 [1.11, 4.95]
Kintwa et al 2021	15	91	4	49	5.2%	2.22 [0.69, 7.10]
Talbert et al 2012	49	142	73	450	7.8%	2.72 [1.77, 4.17]
Wen et al 2021	51	192	76	512	7.9%	2.08 [1.39, 3.10]
Subtotal (95% CI)		1418		1789	44.0%	3.02 [2.16, 4.23]
Total events	264		182			
Heterogeneity: $\mathrm{Tau}^2 = 0.07$; $\mathrm{Chi}^2 = 9.88$, df = 6 ($P = 0.13$); $I^2 = 39\%$						
Test for overall effect: $Z = 6.46$ ($P < 0.00001$)						
10.1.2 General						
Akinbami et al 2010	8	51	3	113	4.5%	6.82 [1.73, 26.92]
Berkley et al 2005	166	873	193	7345	8.3%	8.70 [6.97, 10.86]
Dramaix et al 1993	57	175	105	868	8.0%	3.51 [2.41, 5.11]
Sachdeva et al 2016	41	219	83	1444	7.9%	3.78 [2.52, 5.67]
Sylla et al 2015	15	186	24	246	7.0%	0.81 [0.41, 1.59]
Subtotal (95% CI)		1504		10016	35.7%	3.59 [1.69, 7.64]
Total events	287		408			
Heterogeneity: $\mathrm{Tau}^2 = 0.63$; $\mathrm{Chi}^2 = 58.00$, df = 4 ($P < 0.00001$); $I^2 = 93\%$						
Test for overall effect: $Z = 3.32$ ($P = 0.0009$)						
10.1.3 Other						
Eckerle et al 2022	11	619	10	265	6.3%	0.46 [0.19, 1.10]
Gallagher et al 2023	40	304	33	1760	7.7%	7.93 [4.91, 12.80]
Macpherson et al 2019	8	47	35	685	6.4%	3.81 [1.66, 8.77]
Subtotal (95% CI)		970		2710	20.3%	2.47 [0.47, 13.07]
Total events	59		78			
Heterogeneity: $\mathrm{Tau}^2 = 2.02$; $\mathrm{Chi}^2 = 32.47$, df = 2 ($P < 0.00001$); $I^2 = 94\%$						
Test for overall effect: $Z = 1.06$ ($P = 0.29$)						
Total (95% CI)		3892		14515	100.0%	3.22 [2.12, 4.91]
Total events	610		668			
Heterogeneity: $\mathrm{Tau}^2 = 0.54$; $\mathrm{Chi}^2 = 120.64$, df = 14 ($P < 0.00001$); $I^2 = 88\%$						
Test for overall effect: $Z = 5.46$ ($P < 0.00001$)						
Test for subgroup differences: $\mathrm{Chi}^2 = 0.24$, df = 2 ($P = 0.89$), $I^2 = 0\%$						

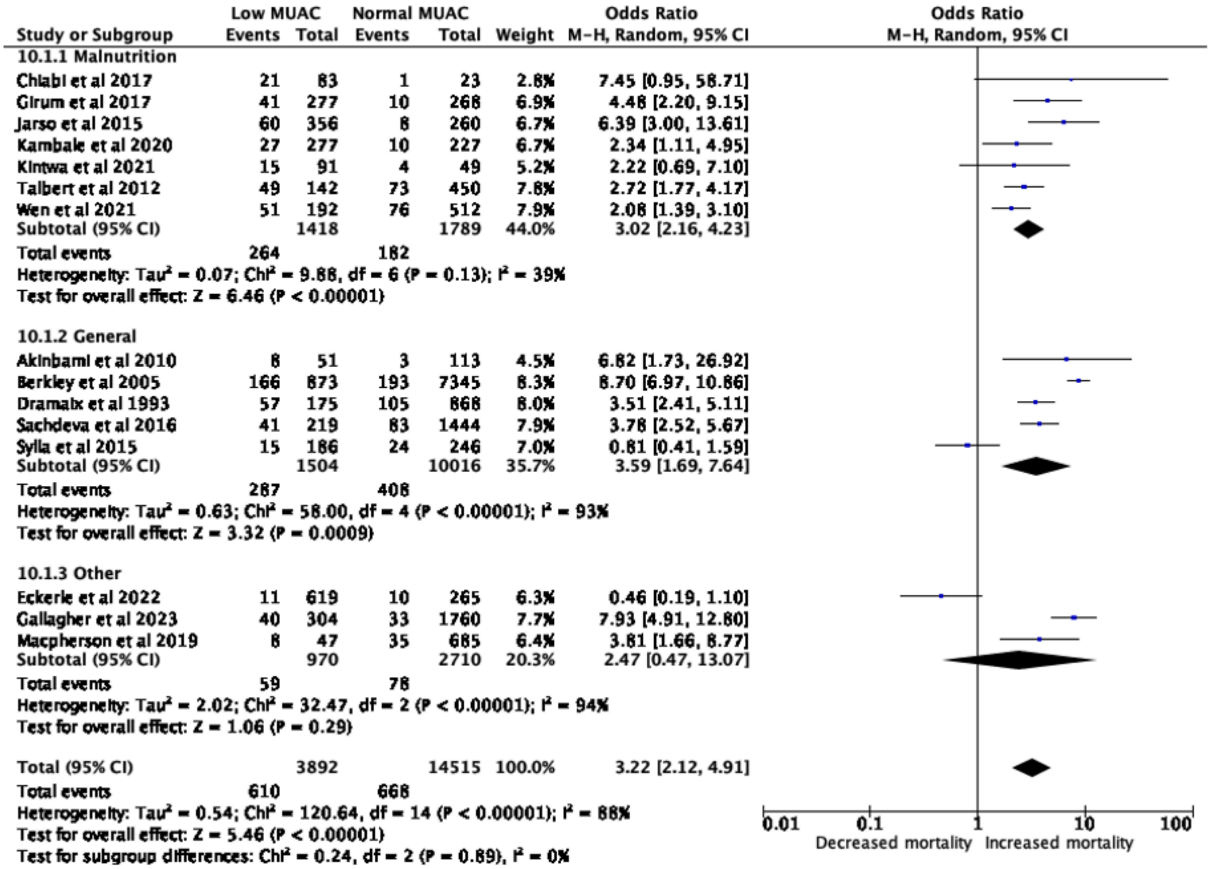

b. WHZ

Study or Subgroup	Low WHZ Events	Low WHZ Total	Normal WHZ Events	Normal WHZ Total	Weight	Odds Ratio M-H, Random, 95% CI
**11.1.1 General**						
Akinbami et al 2010	2	22	9	142	2.1%	1.48 [0.30, 7.34]
Berkley et al 2005	91	756	208	7432	10.4%	4.75 [3.67, 6.16]
Chimhuya et al 2007	70	125	27	112	7.4%	4.01 [2.29, 7.01]
Kumar et al 2020	44	443	39	1260	8.6%	3.45 [2.21, 5.39]
Ochora et al 2024	7	79	9	129	4.0%	1.30 [0.46, 3.63]
Sachdeva et al 2016	44	372	80	1291	9.1%	2.03 [1.38, 2.99]
Sylla et al 2015	27	117	12	315	6.0%	7.58 [3.69, 15.56]
Subtotal (95% CI)		1914		10681	47.6%	3.33 [2.24, 4.95]
Total events	285		384			
Heterogeneity: $Tau^2 = 0.18; Chi^2 = 22.18, df = 6 (P = 0.001); I^2 = 73\%$						
Test for overall effect: Z = 5.97 (P < 0.00001)						
**11.1.2 Other**						
Adegoke et al 2012	11	44	18	267	5.2%	4.61 [2.00, 10.61]
Chiabi et al 2017	19	85	3	21	2.8%	1.73 [0.46, 6.50]
Chisti et al 2011-1	9	61	20	197	5.1%	1.53 [0.66, 3.57]
Demers et al 2000	12	48	31	327	5.8%	3.18 [1.50, 6.74]
Kapoor et al 2022	12	63	5	117	3.7%	5.27 [1.76, 15.75]
Kuti et al 2013	8	77	7	312	3.9%	5.05 [1.77, 14.40]
Lindtjorn et al 1991	12	23	9	82	3.8%	8.85 [3.03, 25.84]
Njuguna et al 2019	18	64	13	117	5.4%	3.13 [1.42, 6.92]
Orimadegun et al 2014	20	140	10	229	5.5%	3.65 [1.65, 8.05]
Shahunja et al 2013	6	41	17	110	4.1%	0.94 [0.34, 2.57]
Sigauque et al 2009	20	115	43	538	7.3%	2.42 [1.36, 4.30]
Subtotal (95% CI)		761		2317	52.4%	3.04 [2.17, 4.25]
Total events	147		176			
Heterogeneity: $Tau^2 = 0.12; Chi^2 = 15.94, df = 10 (P = 0.10); I^2 = 37\%$						
Test for overall effect: Z = 6.50 (P < 0.00001)						
**Total (95% CI)**		2675		12998	100.0%	3.19 [2.47, 4.11]
Total events	432		560			
Heterogeneity: $Tau^2 = 0.14; Chi^2 = 39.84, df = 17 (P = 0.001); I^2 = 57\%$						
Test for overall effect: Z = 8.95 (P < 0.00001)						
Test for subgroup differences: $Chi^2 = 0.12, df = 1 (P = 0.73), I^2 = 0\%$						

Odds Ratio
M-H, Random, 95% CI

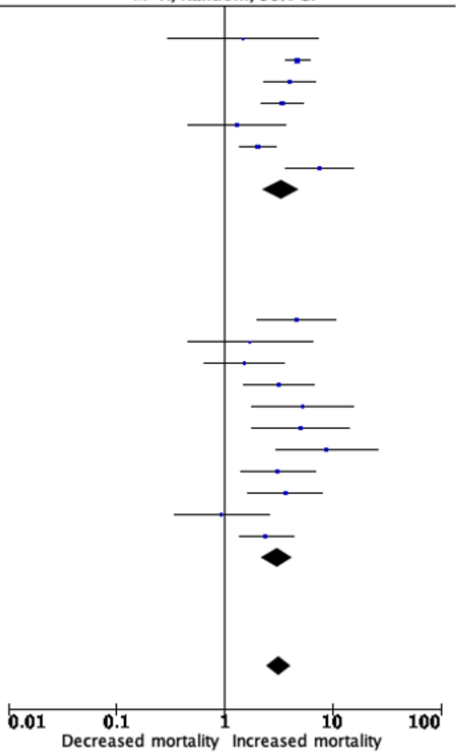

OR, Odds Ratio; CI, Confidence Interval.
