# Supplementary material for: Predicting in-hospital mortality in children in low- and middle-income countries: A systematic review and meta-analysis of vital signs and anthropometric measurements
Source: PLoS One. 2025 Nov 10;20(11):e0336233. doi: 10.1371/journal.pone.0336233 (PMC12599941; doi:10.1371/journal.pone.0336233)
Supplement: S3 Table — (PDF) [file pone.0336233.s008.pdf]

S3 Table. Risk of bias assessment (Newcastle Ottawa Scale)

				Newcastle Ottawa Scale			
Author	Year	Country	Study design	Selection	Comparability	Outcome/Exposure	Total
Abdel Baseer	2022	Egypt	Prospective Cohort	4	1	3	8/9
Abdulkadir	2015	Nigeria	Cross-sectional	5	1	3	9/10
Abrar	2016	India	Prospective cohort	3	0	3	6/9
Adegoke	2012	Nigeria	Cross-sectional	4	0	3	7/10
Adejuyighe	1996	Nigeria	Prospective cohort	4	0	3	7/9
Agweyu	2018	Kenya	Retrospective cohort	4	2	3	9/9
Ahmed Ali	2017	Sudan	Retrospective cohort	4	0	3	7/9
Ahmed	2011	Pakistan	Cross-sectional	4	0	3	7/10
Airlangga	2024	Indonesia	Retrospective Cohort	4	1	3	8/9
Akinbami	2010	Nigeria	Prospective cohort	4	2	3	9/9
Alam	2023	India	Prospective + Retrospective Cohort	4	2	3	9/9
Alao	2023	Nigeria	Prospective cohort	4	2	2	8/9
Alege	2024	Nigeria	Prospective Cohort	4	2	3	9/9
Awasthi	2023	India	Prospective cohort	4	2	2	8/9

Bains	2012	India	Prospective cohort	4	1	3	8/9
Bashaka	2019	Tanzania	Prospective cohort	4	0	3	7/9
Berkley	2005	Kenya	Prospective cohort	4	2	3	9/9
Berkley	2003	Kenya	Prospective cohort	4	2	3	9/9
Bokade	2014	India	Prospective cohort	2	2	2	6/9
Brady	1996	Zimbabwe	Prospective cohort	4	0	2	6/9
Briend	1986	Bangladesh	Prospective cohort	4	2	3	9/9
Chiabi	2016	Cameroon	Case-control	4	1	3	8/9
Chimhuya	2007	Zimbabwe	Cross-sectional	4	0	2	6/10
Chisti	2012	Bangladesh	Prospective cohort	4	2	2	8/9
Chisti	2011-1	Bangladesh	Prospective cohort	4	2	2	8/9
Chisti	2011-2	Bangladesh	Prospective cohort	4	2	2	8/9
Dembele	2019	Philippines	Case-control	4	2	3	9/9
Demers	2000	Central African Republic	Prospective cohort	4	2	3	9/9
Djelantik	2003	Indonesia	Retrospective cohort	4	2	3	9/9
Dramaix	1993	Congo	Prospective cohort	4	2	3	9/9
Duke	2001	Papua New Guinea	Prospective cohort	4	1	3	8/9

Eckerle	2022	Malawi	Prospective cohort	4	0	1	5/9
Ekoube	2024	Cameroon	Retrospective Cohort	4	2	3	9/9
Fattahi	2022	Iran	Retrospective Cohort	4	2	3	9/9
Fouad	2011	Egypt	Prospective cohort	4	2	3	9/9
Gachau	2018	Kenya	Retrospective cohort	4	0	3	7/9
Gallagher	2023	Kenya, Zambia, South Africa, Mali, Gambia, Bangladesh, Thailand	Retrospective Cohort	4	2	3	9/9
George	2015	Kenya, Uganda and Tanzania	Prospective cohort	4	0	3	7/9
Girum	2018	Ethiopia	Retrospective cohort	4	2	3	9/9
Girum	2017	Ethiopia	Retrospective cohort	4	2	3	9/9
Graham	2019	Nigeria	Prospective cohort	4	2	3	9/9
Gupta	2023	India	Retrospective Cohort	4	2	3	9/9
Ikobah	2022	Nigeria	Prospective cohort	3	2	2	7/9
Ilunga-Ilunga	2014	Congo	Prospective cohort	4	2	2	8/9
Jarso	2015	Ethiopia	Retrospective cohort	4	2	3	9/9

Jofiro	2018	Ethiopia	Retrospective cohort	4	2	2	8/9
Jung	2009	Korea	Retrospective cohort	4	1	3	8/9
Kambale	2019	Congo	Retrospective cohort	4	2	3	9/9
Kapoor	2022	India	Case-control	4	2	2	8/9
Kassaw	2021	Ethiopia	Retrospective cohort	4	2	3	9/9
Kintwa	2021	Papua New Guinea	Prospective cohort	4	0	3	7/9
Kouéta	2007	Burkina Faso	Case-control	4	2	2	8/9
Kumar	2020	India	Prospective cohort	4	0	3	7/9
Kumar	2003	India	Prospective cohort	4	1	3	8/9
Kuti	2013	Gambia	Prospective cohort	4	2	3	9/9
Lazzerini	2016	Malawi	Retrospective cohort	4	2	3	9/9
Lindtjorn	1991	Ethiopia	Retrospective case-control	4	0	3	7/9
Macpherson	2019	Kenya	Retrospective cohort	4	2	3	9/9
Maitland	2006	Kenya	Retrospective cohort	4	1	3	8/9
Marazzi	2014	Mozambique, Malawi, Guinea	Retrospective cohort	4	2	3	9/9
Mishra	2023	India	Prospective Cohort	4	0	3	7/9

Muhanuzi	2019	Tanzania	Prospective cohort	4	0	3	7/9
Mujuru	2012	Zimbabwe	Prospective cohort	4	0	2	6/9
Muoneke	2011	Nigeria	Cross-sectional	4	1	3	8/10
Nakubeera-Barungi	2018	Uganda	Prospective cohort	4	2	3	9/9
Nantanda	2008	Uganda	Prospective cohort	4	1	2	7/9
Nantanda	2014	Uganda	Prospective cohort	4	1	2	7/9
Nasir	2011	Nigeria	Retrospective cohort	3	1	2	6/9
Nathoo	1998	Zimbabwe	Retrospective cohort	4	1	2	7/9
Ngaboyeka	2023	Congo	Retrospective cohort	4	2	3	9/9
Nguyen	2022	Vietnam	Retrospective cohort	4	0	3	7/9
Njuguna	2019	Kenya	Retrospective cohort	4	0	3	7/9
Ochora	2024	Uganda	Prospective Cohort	4	2	3	9/9
Odeyemi	2021	Nigeria	Prospective cohort	4	2	3	9/9
Olson	2013	Malawi	Case-control	3	0	2	5/9
Olupot-Olupot	2020	Uganda	Prospective cohort	4	1	2	7/9
Orimadegun	2014	Nigeria	Cross-sectional	4	2	2	8/10
Pannell	2014	Afghanistan	Retrospective cohort	4	0	3	7/9

Rahman	2021	Bangladesh	Retrospective cohort	4	2	3	9/9
Ramakrishna	2012	Malawi	Prospective cohort	4	2	3	9/9
Roy	2011	Bangladesh	Case-control	3	2	1	6/9
Sachdeva	2016	India	Cross-sectional	5	0	3	8/10
Samuel	2014	Malawi	Retrospective cohort	4	0	3	7/9
Schellenberg	1999	Tanzania	Retrospective cohort	4	1	3	8/9
Shafaei	2023	Iran	Case-control	4	1	3	8/9
Shah	2016	Sierra Leone	Retrospective cohort	4	2	3	9/9
Shahunja	2013	Bangladesh	Prospective cohort	4	2	3	9/9
Shahunja	2020	Bangladesh	Retrospective cross-sectional	4	2	3	9/9
Shann	1989	Papua New Guinea	Prospective cohort	4	0	1	5/9
Sharma	2021	India	Retrospective cohort	4	2	3	9/9
Sigauque	2009	Mozambique	Prospective cohort	4	1	1	6/9
Smyth	1997	Zambia	Prospective cohort	4	1	3	8/8
Spooner	1998	Papua New Guinea	Prospective cohort	3	0	2	5/9
Sturgeon	2023	Zimbabwe, Zambia	Prospective Cohort	4	2	1	7/9
Sylla	2015	Senegal	Retrospective cohort	4	2	3	9/9

Talabi	2014	Nigeria	Retrospective cohort	4	2	3	9/9
Talbert	2009	Kenya	Prospective cohort	4	0	3	7/9
Talbert	2012	Kenya	Prospective descriptive	4	2	3	9/9
Talbert	2019	Kenya	Retrospective cohort	4	2	3	9/9
Tette	2016	Ghana	Case-control	3	2	1	6/9
Tuti	2017	Kenya	Retrospective cohort	3	2	3	8/9
Van den Broek	2005	Bangladesh	Case-control	2	2	2	6/9
Waller	1995	Gambia	Prospective cohort	4	2	2	8/9
Wen	2021	Kenya/Malawi	Prospective cohort	4	0	3	7/9
				3.90	1.30	2.66	7.85
