# Supplementary material for: Predicting in-hospital mortality in children in low- and middle-income countries: A systematic review and meta-analysis of vital signs and anthropometric measurements
Source: PLoS One. 2025 Nov 10;20(11):e0336233. doi: 10.1371/journal.pone.0336233 (PMC12599941; doi:10.1371/journal.pone.0336233)

S5 Fig. Funnel plots publication bias

a. Hypoxaemia

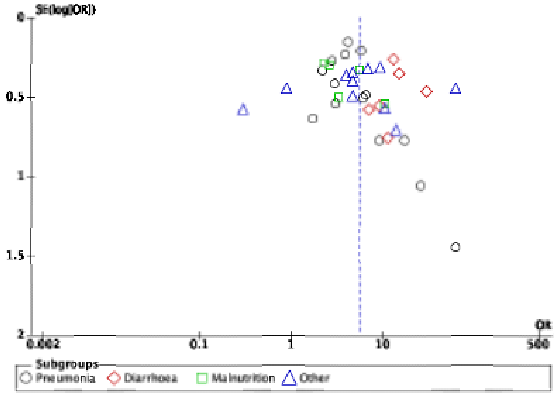

b. Hypoxaemia (cut-off values)

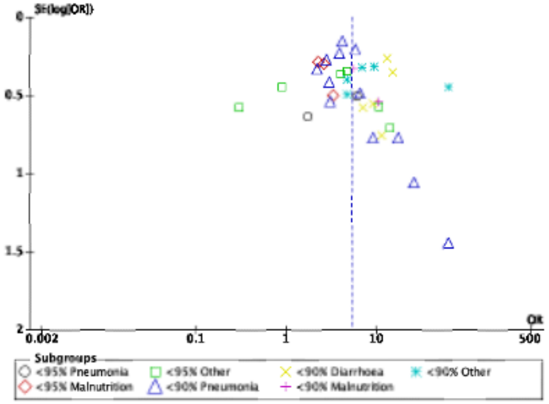

c. Tachypnoea

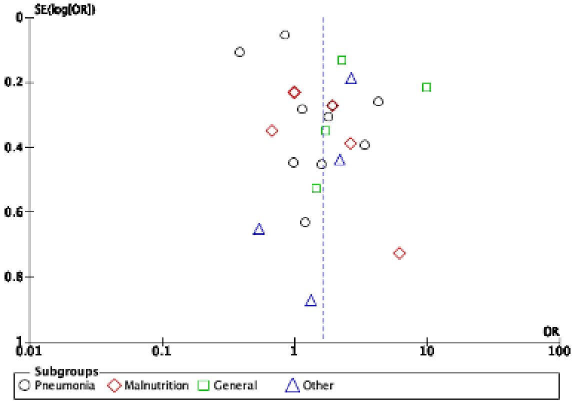

d. Bradypnoea

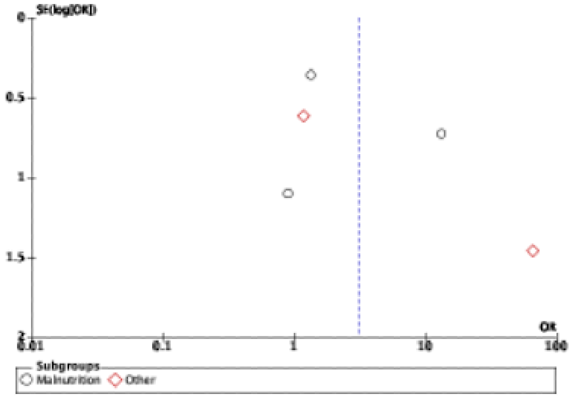

e. Tachycardia

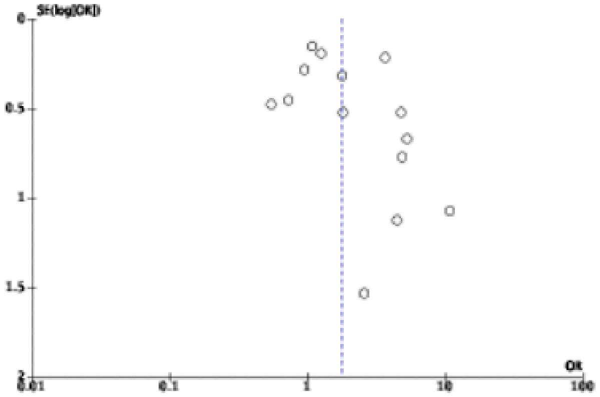

f. Bradycardia

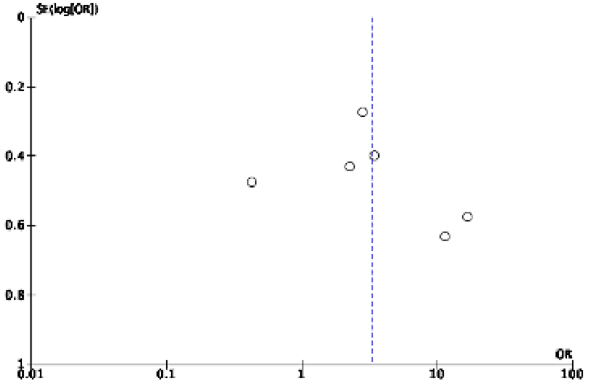

g. Hypertension

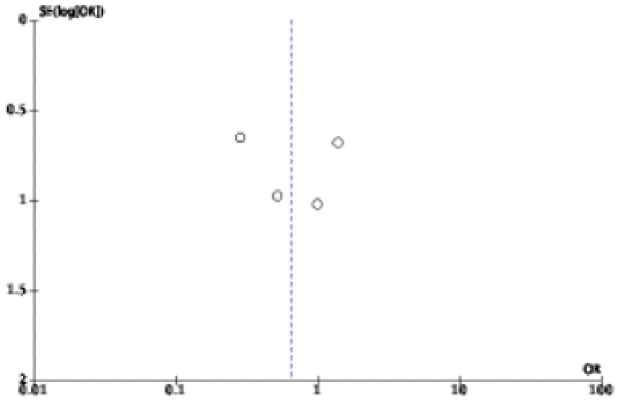

h. Hypotension

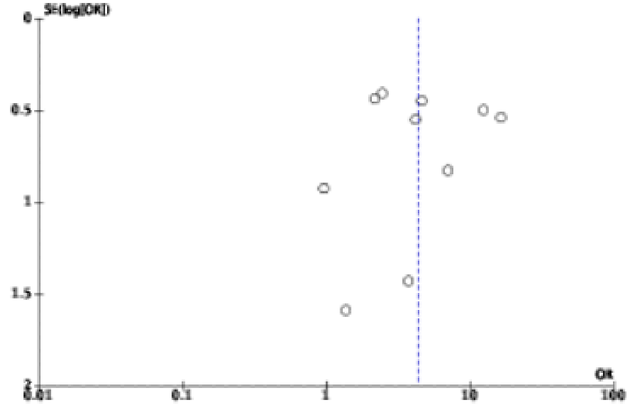

i. Hyperthermia

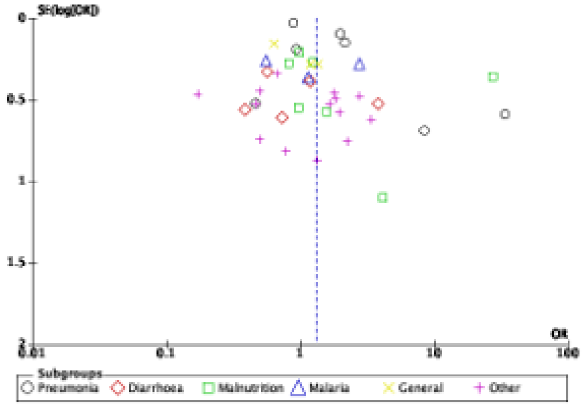

j. Hypothermia

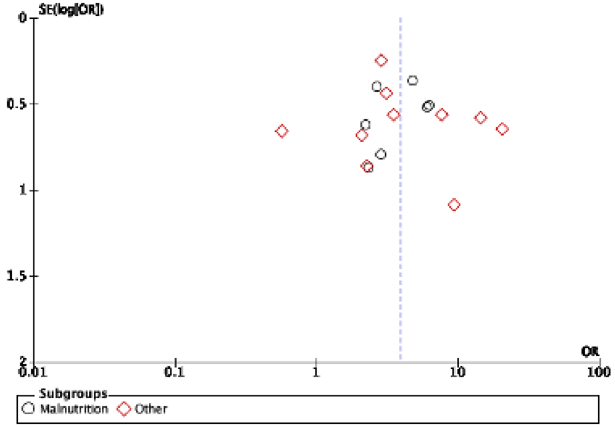

k. Low MUAC

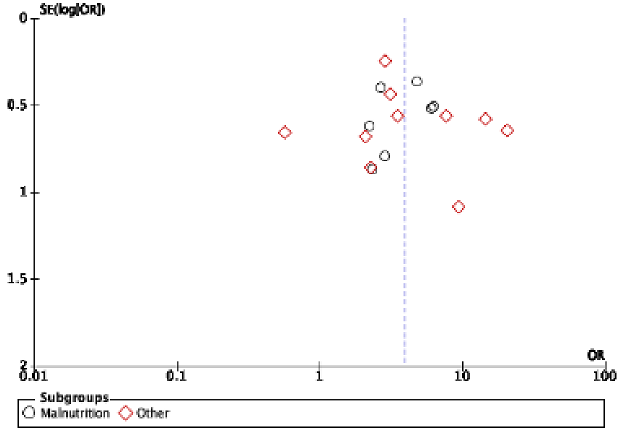

l. Low WHZ

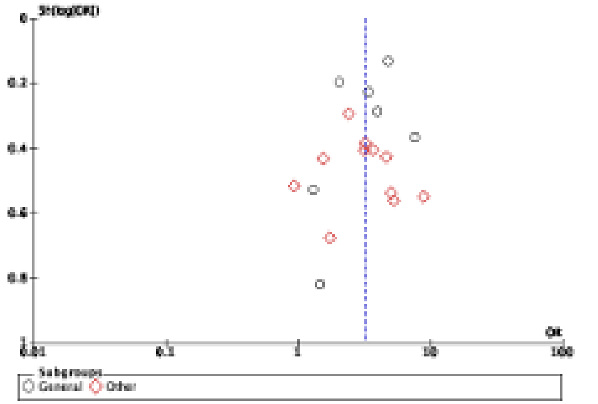

Supplement: S5 Fig — (PDF) [file pone.0336233.s009.pdf]
