# Supplementary material for: Predicting in-hospital mortality in children in low- and middle-income countries: A systematic review and meta-analysis of vital signs and anthropometric measurements
Source: PLoS One. 2025 Nov 10;20(11):e0336233. doi: 10.1371/journal.pone.0336233 (PMC12599941; doi:10.1371/journal.pone.0336233)
Supplement: S4 Table — (PDF) [file pone.0336233.s010.pdf]

S4 Table. GRADE assessment

a. Hypoxaemia

GRADE criteria	Rating	Footnotes	Quality of the Evidence
**Outcome: In-hospital child mortality**			**= Low-Moderate**
Study Design	Low quality	Non-RCT	
Risk of Bias	High quality	Majority of the studies had low risk of bias	
Inconsistency	Very low quality	Considerable heterogeneity between studies	
Indirectness	Low quality	Population’s age, admission diagnosis, time of measurement, cut-off values	
Imprecision	Moderate quality	Large enough sample size; confidence intervals include moderately meaningful effects	
Publication bias	High quality	Undetected	
Other	NA	NA	

b. Tachypnoea

GRADE criteria	Rating	Footnotes	Quality of the Evidence
**Outcome: In-hospital child mortality**			**= Low-Moderate**
Study Design	Low quality	Non-RCT	
Risk of Bias	High quality	Majority of the studies had low risk of bias	
Inconsistency	Very low quality	Considerable heterogeneity between studies	
Indirectness	Low quality	Population's age, admission diagnosis, time of measurement, cut-off values	
Imprecision	Moderate quality	Large enough sample size; confidence intervals include moderately meaningful effects	
Publication bias	High quality	Undetected	
Other	NA	NA	

c. Bradypnoea

GRADE criteria	Rating	Footnotes	Quality of the Evidence
**Outcome: In-hospital child mortality**			**= Low**
Study Design	Low quality	Non-RCT	
Risk of Bias	High quality	Majority of the studies had low risk of bias	
Inconsistency	Low quality	Substantial heterogeneity between studies	
Indirectness	Low quality	Population's age, admission diagnosis, time of measurement, cut-off values	
Imprecision	Very low quality	Small sample size; confidence intervals does not include meaningful effects	
Publication bias	Low quality	All results from small studies, skewed funnel plot	
Other	NA	NA	

d. Tachycardia

GRADE criteria	Rating	Footnotes	Quality of the Evidence
**Outcome: In-hospital child mortality**			**= Low-Moderate**
Study Design	Low quality	Non-RCT	
Risk of Bias	High quality	Majority of the studies had low risk of bias	
Inconsistency	Low quality	Substantial heterogeneity between studies	
Indirectness	Low quality	Population's age, admission diagnosis, time of measurement, cut-off values	
Imprecision	Moderate quality	Large enough sample size; confidence intervals include moderately meaningful effects	
Publication bias	High quality	Undetected	
Other	NA	NA	

e. Bradycardia

GRADE criteria	Rating	Footnotes	Quality of the Evidence
**Outcome: In-hospital child mortality**			**= Low**
Study Design	Low quality	Non-RCT	
Risk of Bias	Moderate quality	75% of the studies low risk of bias, 25% of the studies moderate risk of bias	
Inconsistency	Very low quality	Considerable heterogeneity between studies	
Indirectness	Low quality	Population's age, admission diagnosis, time of measurement, cut-off values	
Imprecision	Low quality	Large enough sample size; confidence intervals include some meaningful effects	
Publication bias	High quality	Undetected	
Other	NA	NA	

f. Hypertension

GRADE criteria	Rating	Footnotes	Quality of the Evidence
**Outcome: In-hospital child mortality**			**= Low-Moderate**
Study Design	Low quality	Non-RCT	
Risk of Bias	High quality	Majority of the studies had low risk of bias	
Inconsistency	High quality	Low heterogeneity between studies	
Indirectness	Low quality	Population's age, admission diagnosis, time of measurement, cut-off values	
Imprecision	Very low quality	Small sample size; confidence intervals does not include meaningful effects	
Publication bias	Moderate quality	All results from small studies	
Other	NA	NA	

g. Hypotension

GRADE criteria	Rating	Footnotes	Quality of the Evidence
**Outcome: In-hospital child mortality**			**= Moderate**
Study Design	Low quality	Non-RCT	
Risk of Bias	High quality	Majority of the studies had low risk of bias	
Inconsistency	Moderate quality	Moderate heterogeneity between studies	
Indirectness	Low quality	Population's age, admission diagnosis, time of measurement, cut-off values	
Imprecision	Moderate quality	Large enough sample size; confidence intervals include some meaningful effects	
Publication bias	High quality	Undetected	
Other	NA	NA	

h. Hyperthermia

GRADE criteria	Rating	Footnotes	Quality of the Evidence
**Outcome: In-hospital child mortality**			**= Low-Moderate**
Study Design	Low quality	Non-RCT	
Risk of Bias	High quality	Majority of the studies had low risk of bias	
Inconsistency	Very low quality	Considerable heterogeneity between studies	
Indirectness	Low quality	Population's age, admission diagnosis, time of measurement, cut-off values	
Imprecision	High quality	Large enough sample size; confidence intervals include meaningful effects	
Publication bias	High quality	Undetected	
Other	NA	NA	

i. Hypothermia

GRADE criteria	Rating	Footnotes	Quality of the Evidence
**Outcome: In-hospital child mortality**			**= Low-Moderate**
Study Design	Low quality	Non-RCT	
Risk of Bias	High quality	Majority of the studies had low risk of bias	
Inconsistency	Low quality	Substantial heterogeneity between studies	
Indirectness	Low quality	Population's age, admission diagnosis, time of measurement, cut-off values	
Imprecision	Moderate quality	Large enough sample size; confidence intervals include moderately meaningful effects	
Publication bias	High quality	Undetected	
Other	NA	NA	

j. MUAC

GRADE criteria	Rating	Footnotes	Quality of the Evidence
**Outcome: In-hospital child mortality**			**= Low-Moderate**
Study Design	Low quality	Non-RCT	
Risk of Bias	High quality	Majority of the studies had low risk of bias	
Inconsistency	Very low quality	Considerable heterogeneity between studies	
Indirectness	Low quality	Population's age, admission diagnosis, time of measurement, cut-off values	
Imprecision	Moderate quality	Large enough sample size; confidence intervals include moderately meaningful effects	
Publication bias	High quality	Undetected	
Other	NA	NA	

k. WHZ

GRADE criteria	Rating	Footnotes	Quality of the Evidence
**Outcome: In-hospital child mortality**			**= Low-Moderate**
Study Design	Low quality	Non-RCT	
Risk of Bias	High quality	Majority of the studies had low risk of bias	
Inconsistency	Low quality	Substantial heterogeneity between studies	
Indirectness	Low quality	Population's age, admission diagnosis, time of measurement, cut-off values	
Imprecision	Moderate quality	Large enough sample size; confidence intervals include moderately meaningful effects	
Publication bias	High quality	Undetected	
Other	NA	NA	
